# Metabolic response induced by parasitic plant-fungus interactions hinder amino sugar and nucleotide sugar metabolism in the host

**DOI:** 10.1038/srep37434

**Published:** 2016-11-28

**Authors:** Dong-Kyu Lee, Soohyun Ahn, Hae Yoon Cho, Hye Young Yun, Jeong Hill Park, Johan Lim, Jeongmi Lee, Sung Won Kwon

**Affiliations:** 1 College of Pharmacy, Seoul National University, Seoul 08826, Korea; 2 Department of Statistics, Seoul National University, Seoul 08826, Korea; 3 Department of Agricultural Biotechnology, College of Agriculture and Life Sciences, Seoul National University, Seoul 08826, Korea; 4 Research Institute of Pharmaceutical Sciences, Seoul National University, Seoul 08826, Korea; 5 Faculty of Pharmacy, Ton Duc Thang University, Ho Chi Minh City, Vietnam; 6 School of Pharmacy, Sungkyunkwan University, Suwon 16419, Korea; 7 Plant Genomics and Breeding Institute, Seoul National University, Seoul 08826, Korea

## Abstract

Infestation by the biotrophic pathogen *Gymnosporangium asiaticum* can be devastating for plant of the family Rosaceae. However, the phytopathology of this process has not been thoroughly elucidated. Using a metabolomics approach, we discovered the intrinsic activities that induce disease symptoms after fungal invasion in terms of microbe-induced metabolic responses. Through metabolic pathway enrichment and mapping, we found that the host altered its metabolite levels, resulting in accumulation of tetrose and pentose sugar alcohols, in response to this fungus. We then used a multiple linear regression model to evaluate the effect of the interaction between this abnormal accumulation of sugar alcohol and the group variable (control/parasitism). The results revealed that this accumulation resulted in deficiency in the supply of specific sugars, which led to a lack of amino sugar and nucleotide sugar metabolism. Halting this metabolism could hamper pivotal functions in the plant host, including cell wall synthesis and lesion repair. In conclusion, our findings indicate that altered metabolic responses that occur during fungal parasitism can cause deficiency in substrates in pivotal pathways and thereby trigger pathological symptoms.

Parasitism has undoubtedly contributed significantly to human life, and researchers have according investigated parasitic fungal pathology, with a particular focus on biological phenomena. Basic symptoms, from initial fungal contact to the establishment of a stable relationship with the host, have been reported^1^. In the wake of such visual assessments, many studies are now addressing gene expression and functional enzymatic reactions related to nutrient exchange as well as the mechanisms of interaction^2^,^3^,^4^. In particular, many studies focus on chemical or biological causal agents released from the invading organism because these molecules initiate plant-fungus interactions. Accordingly, how fungi behave when they contact and interact with their host has been well studied.

The causal agent of rust disease is one such parasite that can have devastating effects on important crops such as apple and pear. Fungi of the genus *Gymnosporangium*, which exhibit heteroecious parasitism, are known to cause rust disease. These fungi produce spores and form yellow or brown galls composed of teliospores on leaves or twigs of junipers, its host during winter. After the warm rain that occurs in spring, gelatinous tendrils comprised of diploidy basidiospore are produced and dispersed into the air. The released spores parasitize Rosaceae species, causing defoliation, a lack of photosynthesis and necrotic lesions. This entire parasitic life cycle has been revealed by many studies, followed by the development of widespread preventions and remedies for preventing rust disease.

However, despite the many rust disease pathologies discovered to date, alterations in biochemical composition in the plant host caused by rust disease have not been thoroughly investigated, and parasitic fungal factors have, in many cases, been considered the cause of host death. Nonetheless, similar to allergic reactions in humans, plant death might result from its own sensitive response to the fungus. In other words, the intracellular response to the invading organism might be a main factor that blocks the natural interaction between the plant and fungus. The complexities of the pathology, which is detrimental to plant, have not been sufficiently explored to verify this hypothesis. Therefore, biochemical analysis of the plant host will aid in our understanding of unknown mechanisms of fungal-induced pathology.

To examine how parasitism alters the host, we used a metabolomics approach to investigate terminal states directly related to fungal-induced pathology. Parasites naturally change the metabolism and biological functions of their host while acquiring specific benefits^5^,^6^,^7^. Accordingly, clues to the underlying pathological or virulent pathways may be found by assessing changes in the abundance of different metabolites, and a metabolomics approach may be valuable for investigating unknown biochemical responses in plant-fungal interactions. In this study, we compared metabolic profiles of control and parasitized leaves and further interpreted complex changes and relationships within the metabolic network of the plant host. Using this approach, we determined that a fungal-induced metabolic strategy can block the normal regulation of amino sugar and nucleotide sugar metabolism and that this phenomenon is capable of causing intrinsic pathology.

## Results

### Parasite-induced metabolic alterations in Rosaceae hosts

We first identified the fungus present on the parasitized leaves of all samples. The pathological manifestations (e.g., fungal aecia and deformities of colored lesions (Supplementary Fig. S1), morphological characteristics (Supplementary Fig. S2) and host range observed in each species) confirmed the rust fungus to be *Gymnosporangium asiaticum*^8^. And for verifying the identification, DNA was isolated from collected aecia using protocols modified from those of Harrington *et al.*^9^. The LSU (large subunit, 28S) rDNA region was amplified with forward primer No. 4 and reverse primer No. 11^10^. PCR products were also sequenced using primers No. 4 and No. 11^11^. For phylogenetic analysis, sequences obtained in this study and sequences of *Gymnosporangium* species in the GenBank sequence database were aligned and compared by pairwise alignment using jPHYDIT^12^. Molecular sequence data from these aecial specimens were confirmed by comparison with previously published South Korean specimens in the GenBank database (FJ848744, FJ848745, FJ848746, 630 bp matching)^11^.

**Figure 1:**
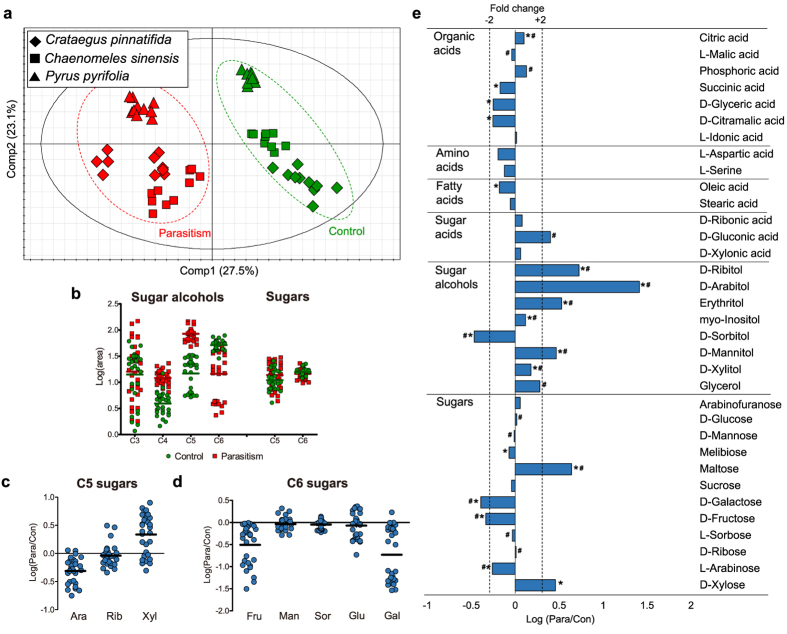
Control and parasitized leaves of Rosaceae differ in metabolite levels. **(a)** PLS-DA score plots of CP (diamond, n = 20), CS (box, n = 20) and PS (triangle, n = 20) under the two conditions (parasitism, red; control, green) with PC1 and PC2. Validation parameters are as follows: R^2^X = 0.565, R^2^Y = 0.963 and Q^2^ = 0.942 **(b)** Scatter column plots of sugar alcohols (C3-C6) and sugars (C5-C6) in control (green) and parasitized (red) leaves divided by carbon number. **(c)** Scatter column plot with log-scale ratios of C5 sugars and **(d)** C6 sugars (the centered line represents the mean). (**b–d**) Sixty samples were assessed, 30 for each group. **(e)** Logarithmic ratios of metabolites (parasitism/control). Notable metabolites are indicated by asterisks (p-value < 0.05) and a hash sign (VIP value > 1.00) and ±2-fold change by a dashed line (n = 30 for each group).

Next, to examine metabolite patterns regulated by this parasite, metabolites were extracted from three species of the Rosaceae family, *Crataegus pinnatifida* (CP), *Chaenomeles sinensis* (CS) and *Pyrus pyrifolia* (PS) (Supplementary Table S1), followed by derivatization and identification. A total of 34 metabolites, including 7 organic acids, 2 amino acids, 2 fatty acids, 3 sugar acids, 8 sugar alcohols and 12 sugars, were profiled using gas chromatography mass spectrometry (GC-MS; Supplementary Table S1). Three principal component analysis (PCA) score plots (n = 10 for each control and parasitized sample from the three species) revealed discriminative metabolic levels in primary metabolites triggered by fungal-induced parasitism in CP (PC1, explaining 47.4%), CS (PC1, explaining 45.9%) and PS (PC1, explaining 54.5%). This was validated by R^2^cum values of 0.751, 0.787 and 0.83 and Q^2^cum values of 0.475, 0.751 and 0.686, respectively (Supplementary Fig. S3). However, PCA performed using all of the data (n = 60) generated PCs that aligned with host species discriminations (Supplementary Fig. S4). Therefore, we used a supervised method, partial least square-discriminant analysis (PLS-DA), to evaluate parasite-specific changes in metabolite that were common to all three host species (Fig. 1a). The score scatter plots classified samples as ‘control’ and ‘parasitized’, with 27.5% of the variability contained in PC1, and were thoroughly validated by a 200x permutation test (y-intercept of −0.0403) and CV-ANOVA (p-value of 1.13·10^−29^)^13^.

Pivotal metabolite alterations associated with parasitism were identified by considering both multivariate (variable importance for projection; VIP) and univariate (p-values) statistical parameters (Supplementary Table S2). Of the 34 metabolites, only those with a VIP value over 1.00 or a p-value below 0.05 were considered as being significantly altered in the parasitized plants. These criteria indicated that 26 metabolites were reliably altered by rust disease; these alterations are presented in a heat-map derived from hierarchical clustering analysis (Supplementary Fig. S5). The data points were averaged to quantitate the alterations and to further elucidate the general changes induced by fungal invasion across the three host species. Among the markers, sugars were considerably altered with maltose (2.86-fold) and xylose (4.37-fold) being elevated in parasitized leaves and galactose (−2.46-fold) and fructose (−2.16-fold) being reduced. However, the most remarkable imbalance between the two groups occurred in sugar alcohols, which showed greater changes. Ribitol (5.31-fold), arabitol (25.83-fold), erythritol (3.37-fold) and mannitol (2.93-fold) were present at higher levels in the presence of the fungus, whereas sorbitol (−2.92-fold) was reduced. These results suggest that a *G. asiaticum*-induced metabolic strategy in the host species may involve changes in sugars and sugar alcohols, which may indicate why the *Gymnosporangium*-host relationship manifests as intrinsically parasitic.

### Sugar and sugar alcohol regulation caused by fungi-induced parasitism

The most conspicuously affected metabolites, sugars and sugar alcohols were re-sorted by carbon numbers and plotted in column-scattered format to reveal changes in metabolite levels (Fig. 1b). C3 sugar alcohols did not exhibit a relationship with parasitic symptoms. However, higher levels of tetrose (C4; erythritol) and pentose (C5; ribitol, arabitol and xylitol) alcohols, which were previously identified as markers based on multivariate (VIP values) and univariate (p-value) statistical parameters, were observed in the parasitism group in all three plant hosts. Conversely, hexose alcohols (C6; mannitol, sorbitol and inositol) were generally reduced in the parasitism group, though mannitol and inositol were increased in certain cases. In the case of sugars, the plot divided by carbon number did not reveal meaningful differences. However, scatter plots categorized by each sugar demonstrated that except for xylose, which increased slightly, all C5 and C6 sugars were decreased (arabinose for C5; fructose and galactose for C6) or remained constant in the parasitized hosts (Fig. 1c and d). Overall, *G. asiaticum* stimulated the hosts to accumulate tetrose and pentose sugar alcohols and to slightly reduce their stores of certain pentose and hexose sugars.

### Metabolic network analysis related to parasitism

**Figure 2:**
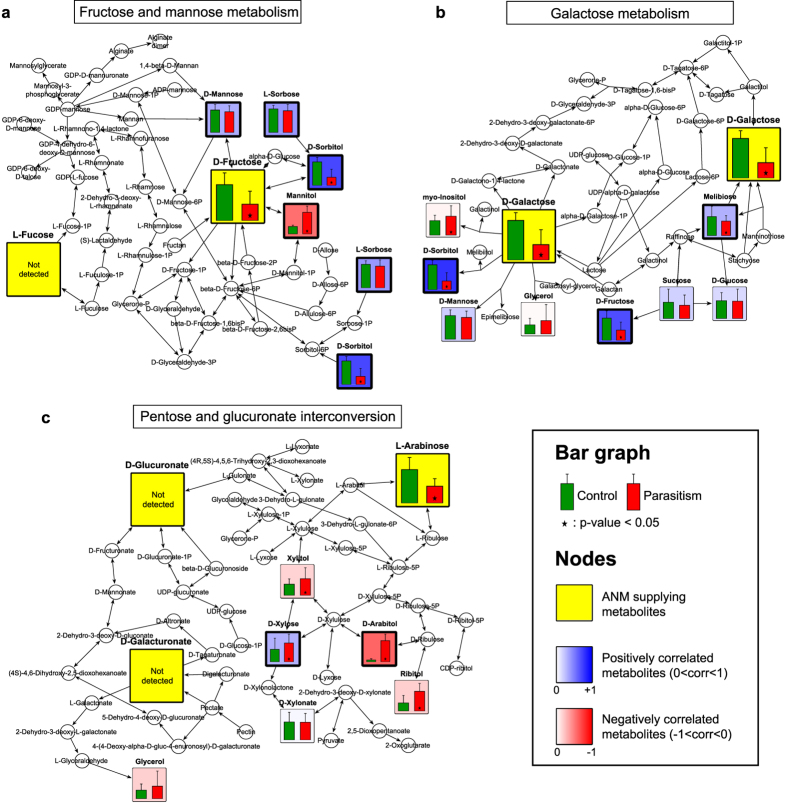
Pathway mapping reveals correlations between ANM supply metabolites and other metabolites. Mapping of metabolic alterations for FMM **(a)**, GM **(b)** and PGI **(c)**. Metabolites (p-value < 0.05, marked with an asterisk) between control (green) and parasitized (red) leaves are colored based on their PPMCC values, ranging from −1 (negative correlation, red) to +1 (positive correlation, blue), against ANM supply metabolites (yellow). Values shown are the mean + S.D., with 30 replicates for each group.

**Figure 3:**
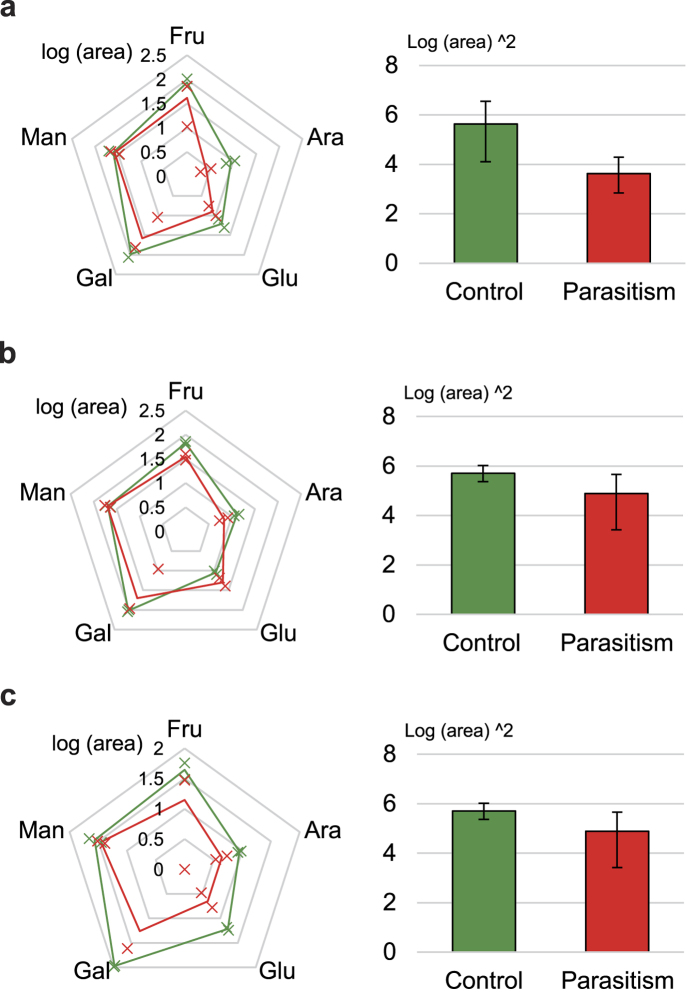
The ANM pathway lacks metabolic substrates under parasitism. Radar charts and pentagonal areas of CP **(a)**, PS **(b)** and CS **(c)**. Five sugars that supply the ANM pathway are denoted with log-scale means ± S.D. (marked by x-shaped points) in the radar charts, and bar graphs show the squared mean and S.D.

Pathway enrichment analysis using the aforementioned 26 metabolite markers suggested possible relationships between these metabolites and biological pathways (Supplementary Table S3). If a pathway had a high matched/total percentage and showed a low adjusted p-value (<0.05), it was considered a candidate related to parasitism. Seven metabolic pathways met these criteria: pentose and glucuronate interconversion (PGI), galactose metabolism (GM), fructose and mannose metabolism (FMM), starch and sucrose metabolism (SSM), glyoxylate and dicarboxylate metabolism (GDM), biosynthesis of plant hormones (BPH) and the tricarboxylic acid cycle (TCA cycle). Next, we attempted to find connections among these pathways. With the exception of SSM and GDM, we found that amino sugar and nucleotide sugar metabolism (ANM) is related to three pathways, (Supplementary Fig. S6). PGI, GM and FMM provide upstream substrates for ANM (glucuronate, galacturonate and arabinose, PGI; fructose, mannose and fucose, FMM; galactose, fructose and mannose, GM). Interestingly, the upstream pathways supply all of the metabolites required for ANM, except for extracellularly supplied N-acetylmuramate and N-acetyl-D-glucosamine, suggesting an important relationship between ANM-related pathways and pathogenic symptoms. To observe possible relationships among the metabolic alterations, pathway mapping of the three ANM-related pathways was subsequently performed using integrated data from the three host species (Fig. 2)^14^. The mapping results showed changes in each metabolite (bar graph). Pearson’s product-moment correlation coefficient (PPMCC) was determined for the ANM supply metabolites, with the exception of certain types of supply metabolites that either did not change reliably (mannose in FMM and GM) or were not found in leaves (fucose in FMM; glucuronate and galacturonate in PGI). Most importantly, alterations in ANM supplies (yellow box) were significantly reduced under parasitism. Pentagonal radar charts showing the levels of five of the ANM supply sugars (excluding those existing under the limit of detection) in healthy and parasitized plants further supported that ANM supplies truly declined during parasitism, though we could not determine specific causes for the decreases in glucose and mannose (Fig. 3). The sizes of the three pentagons clearly decreased under parasitism, indicating that supply deficiency occurred.

Accordingly, we observed that metabolic changes correlated negatively with ANM supplies (red box), indicating that their accumulation possibly affected the decrease in ANM supplies. FMM strongly negatively correlated with fructose reduction and mannitol accumulation (Fig. 2a). In the case of GM, no metabolic changes correlated with the observed reductions in galactose (Fig. 2b), fructose (Supplementary Fig. S7a), glucose (Supplementary Fig. S7b) or mannose (Supplementary Fig. S7c), the metabolites required for ANM. The most significantly affected pathway was PGI (Fig. 2c), which correlated highly with the metabolites, with high fold changes and low p-values (arabitol, xylitol and ribitol), indicating that the reduction in arabinose could be explained by the accumulation of these three compounds. Therefore, the cause of metabolic abnormalities found during parasitism was assessed from the perspective of these three metabolites.

**Figure 4:**
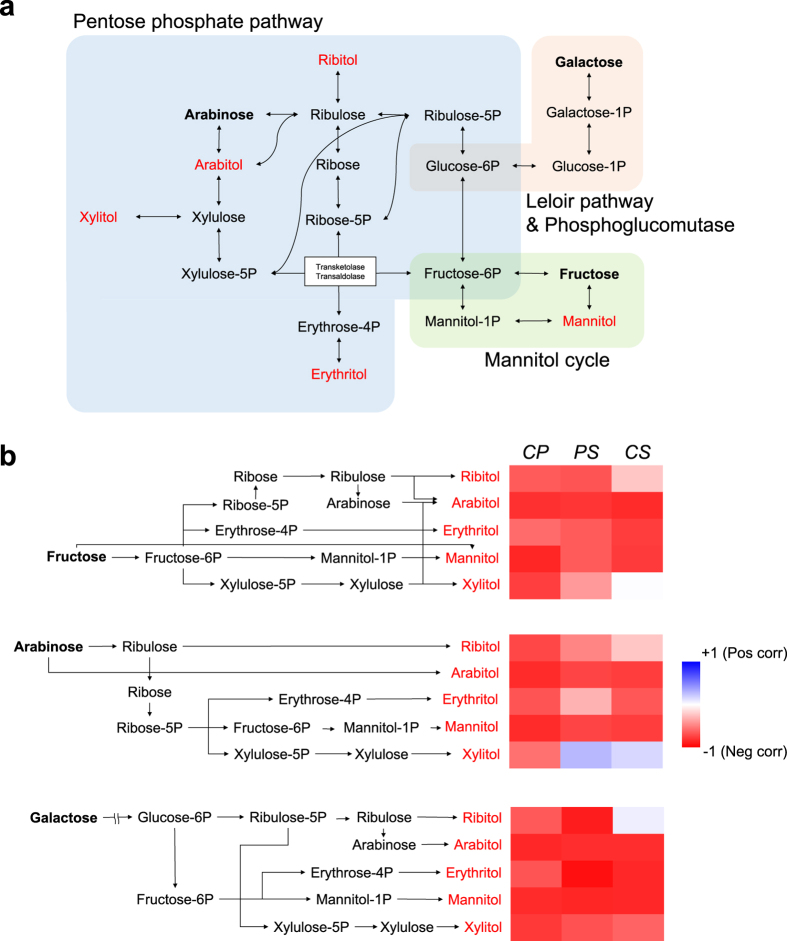
Accumulation of sugar alcohols is related to decreases in ANM upstream metabolites. **(a)** An integrated pathway centered on PPP involving five increased and three decreased metabolites. **(b)** Heat-maps of correlations between metabolic alterations of the two groups based on intermediates of the integrated pathway (the enzymes in each reaction are indicated in Supplementary Table S4). The color depth denotes the degree of correlation; red color represents the opposite alterations and blue color the equivalent alterations under parasitism.

Interestingly, these three metabolites plus erythritol, which was identified as a marker but was not included in the pathway mapping, are indirectly involved in the pentose phosphate pathway (PPP) via their phosphate forms (Fig. 4a). The enzymatic processes of the metabolic network connecting these metabolites, as identified in the KEGG database, highlight their intimate relationships (Supplementary Table S4). Additionally, the mannitol cycle induced by plant-microbe interactions connects mannitol to PPP through its intermediate fructose-6P^15^,^16^. Moreover, galactose is biologically relevant to PPP via the Leloir pathway (galactose to glucose-1P) and phosphoglucomutase (glucose-1P to glucose-6P)^17^,^18^. Thus, a metabolic network including all five metabolites that accumulate under parasitism was reasonably established. To confirm the correlation between metabolic changes in this integrated network, we first acquired the entire metabolite heat-map based on PPMCC measurement (Supplementary Fig. S8) and further selected specific heat-maps for fructose, arabinose and galactose, the three reliably reduced metabolites upstream of ANM. We also generated heat-maps for arabitol, xylitol, ribitol, erythritol and mannitol, the five metabolites that accumulate in the integrated network (Fig. 4b). Although there was a small positive correlation between arabinose and xylitol in PS and CS and between galactose and ribitol in CS, all other relationships showed high absolute values of negative correlation. This signifies a strong regression with the reverse alteration for the accumulation of these metabolites and reductions in the others. In particular, the highest averaged coefficients for each species was found for arabitol: −0.81 (fructose), −0.77 (arabinose) and −0.82 (galactose). This was slightly higher than for mannitol (−0.75, −0.77 and −0.84, respectively), which was previously identified as a common product of plant-fungus interactions. Xylitol showed the fewest correlations with the reduction in ANM supplies (−0.31, −0.07 and −0.56, respectively), but these values still suggest that xylitol contributes to reductions in the other metabolites. With respect to ANM supplies, galactose (−0.73) was most strongly impacted by the accumulation of sugar alcohols, followed by fructose (−0.62) and arabinose (−0.52). These negative correlations provide the basis for a hypothesis regarding the relationship between ANM metabolic deficiency and sugar alcohol accumulation, as discussed below.

### Interaction effects of the control/parasitism variable

Finally, we considered the following multiple linear regression model to explain the effect of interactions between the group variable (control/parasitism) and the levels of sugar alcohols that feed into ANM pathways based on multiple measurements of metabolite levels.


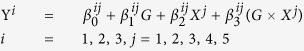


where Y^*i*^ is the ANM supply, with *i* (1:fructose; 2:arabinose; 3:galactose) as the dependent variable, and *G* and *X*^*j*^ are independent variables denoting the group (0: control; 1: parasitism) and the accumulated sugar alcohol *j* (1:ribitol; 2:arabitol; 3:erythritol; 4:mannitol; 5:xylitol). Overall, 45 multiple linear regression models and interaction effect coefficients, 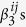, between the group variable and metabolite *j* to metabolite *i* were included in the model (Table 1). The hypothesis regarding the existence of an interaction effect is 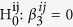. Crossing of regression lines (regression validated by the adjusted R square in Table 1) on the scatter plots crossed owing to a slope difference suggested that the group variable and the sugar alcohols had an interaction effect on the expression of dependent variables (Supplementary Fig. S9). Additionally, the slope test with a min-max normalized dataset (all dependent and independent values were set at 0.00–1.00) explained the effect direction of the group variable (control or parasitism), whereby a minus value indicated that the ANM supply/sugar alcohol ratio decreased under parasitism. Combined with the statistical significance analysis (p < 0.05), this test revealed the effect of sugar alcohols on the dependent variables (ANM supplies). Only the decrease in galactose was shown to be unaffected by the accumulation of sugar alcohols, as only a small number of models (2/15) had a p-value below 0.05; this supports the pathway mapping result that galactose metabolism is not closely related to the accumulation of sugar alcohols. However, considering the number of meaningful models (CP: 6/10, PS: 3/10 and CS: 6/10), the other decreases in fructose and arabinose were significantly related to sugar alcohol accumulation (except for ribitol, which did not cause any decrease in ANM supplies). Furthermore, except for arabitol, all the meaningful models had a negative effect, emphasizing that those sugars and sugar alcohols are closely related in a metabolic network. Consequently, we found a significant interaction effect in some models, representing a significant difference in the levels of dependent metabolites (ANM supplies) between the two groups as levels of the independent metabolites (sugar alcohols) increased.

## Discussion

Interactions between pathogenic fungi and their plant host result in metabolic changes in the latter. We used a metabolomics approach to identify the core metabolic changes caused by the plant-fungus relationship that induce parasitic symptoms. We investigated such metabolic responses in three species of host plants exposed to and dominated by the fungus, after which the reliability of and relationships among metabolic changes were dissected. The results of this investigation first indicated that sugar alcohols accumulate as a plant metabolic response to parasitism. Furthermore, this irregular metabolism causes deficiency in the ANM pathway, which is very important for cell wall synthesis. Because we prepared fungal-infected samples from natural trees rather than from the controlled cultures in our lab, it is possible that some variation existed in our samples. To determine whether the observed variation was significant, we re-collected infected samples from the same trees in the same herbal garden one year later and checked for reproducibility regarding the observed metabolic alterations.

We discovered accumulation of four sugar alcohols; in contrast, previous reports could not demonstrate accumulation of all four sugar alcohols simultaneously or even whether they were the primary cause of deficiency in substrates for the ANM pathway. Accumulation of mannitol and its transformation into fructose were revealed not only by our study but also by previous studies^16^,^19^. With regard to other accumulated sugar alcohols, arabitol has also been identified as a quencher of reactive oxygen species (ROS); the others, erythritol and xylitol, have been reported to accumulate in some infected hosts^20^,^21^,^22^. However, those studies did not consider simultaneous accumulation of these 4 sugar alcohols.

Interestingly, simultaneous accumulation of these metabolites and the relationship between sugar accumulation and deficiency has not been reported to date. In our study, this relationship was revealed by measuring PPMCC. A heat-map of PPMCC showed highly negative correlations between sugars and sugar alcohols (Fig. 4b). Next, to verify that sugar alcohol accumulation affected the reduced levels of metabolites in the ANM pathway, we generated a statistical module of the interaction effect. Multiple linear regression models of fructose and arabinose demonstrated the interaction effect of the combined accumulation of sugar alcohols (sugar alcohols and group variables (control/parasitism)) on dependent variable (upstream sugars in ANM); this deductive logic suggests that the sugar alcohol accumulation induced by the fungal interaction led to the deficiency in ANM substrates. This approach is not commonly applied in metabolomics studies, but it could serve as a useful tool for understanding complex relationships among reactions in a given metabolic network.

**Figure 5:**
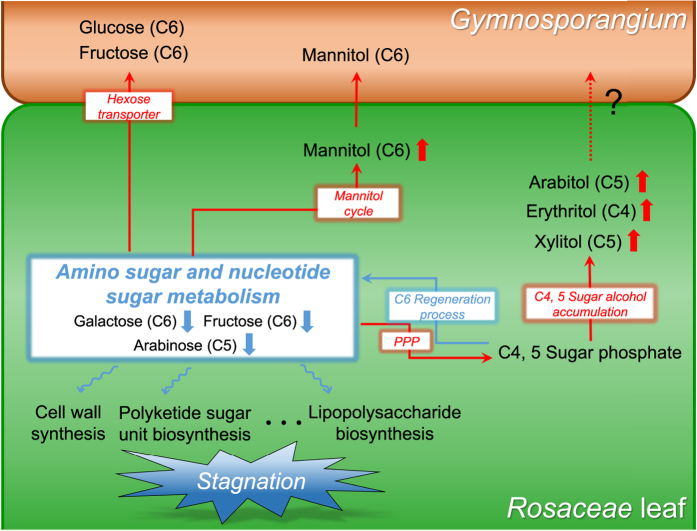
Abnormal accumulation of C4 and C5 sugar alcohols leads to metabolic deficiency in the ANM pathway. Red arrows indicate increases in metabolites or pathway activity; blue arrows indicate the opposite effect.

Together with the effect of sugar alcohol accumulation, many other studies about fungal parasitism support the observed deficiency in ANM substrates (Fig. 5). Our study demonstrates that parasitized hosts have reduced total levels of sugars that serve as upstream substrates for ANM. In addition, many studies have suggested the possible exhaustion of certain upstream metabolites in this pathway. Previous studies have reported that fungal transport of hexose, especially glucose and fructose, are carried out by the hexose transporter (HXT1) present in both mutualistic and parasitic fungi^23^,^24^,^25^,^26^. It is assumed that the reason for this is that fungi prefer hexose as their carbohydrate source. Another study reported possible fructose starvation upon establishment of the plant-fungus interaction because fructose is converted to mannitol. However, these pathways alone could not reveal an association with rust pathology because only a portion of the substrate was shown to decrease or it did not decrease enough to cause supply deficiency in the ANM pathway. When considering our results together with those of previous reports, we conclude that ANM supplies are deficient under plant-fungus interactions, given that simultaneous accumulation of tetrose and pentose sugar alcohols correlated with a decrease in all upstream sugars of the ANM pathway.

Metabolic deficiency of the ANM pathway could impair functional metabolic activities. Indeed, ANM products have many roles in living organisms; in particular, these products are important for maintaining and repairing the cell wall. When a fungus contacts a plant host, the fungus degrades and penetrates the plant cell wall via enhanced cellobiohydrolyase activity to establish haustoria within the host tissue^23^. In response to this penetration, the innate plant defense system in a normal leaf needs to reinforce the destroyed portion of the cell wall. However, callose (1–3 linked β-glucan polysaccharide), which is essential for the defense system, is biologically synthesized from UDP-glucose, a downstream ANM metabolite^27^,^28^,^29^. In addition, fortifying the penetrated lesion with primary and secondary cell wall materials requires sugar units in nucleotide form. Cellulose, the main component of the two types of cell wall, consists of (1–4)-β-glucoside skeletons polymerized from nucleotidyl glucose^30^. Hemicellulose, which connects pectin and cellulose in the two types of cell wall to construct cross-linked fibers in the cell wall network, comprises heteropolymers assembled by many types of pentose (xylose and arabinose) and hexose (galactose, mannose and rhamnose) building blocks with nucleotide diphosphate (NDP)^31^,^32^,^33^. Nucleotide sugars are also required for the biosynthesis of pectin, which is incorporated into the primary cell wall^34^,^35^. Thus, if essential sugar units for producing these components are limiting, the plant host cannot carry out cell wall repair to combat penetration. That is, the metabolic response of the host to the parasite could cause abnormalities in the ANM pathway that contribute to pathogenic symptoms relating to the cell wall.

Several hypotheses could explain why this pathway blockage occurs. The first is that the fungus induces changes in the host’s metabolism for nutrient acquisition or to modify systems for its own benefit, as it is commonly known that certain microbes alter their host for their profit while the host must maintain homeostasis to cope with these changes^36^. Nonetheless, this metabolic problem could paradoxically be caused by the host’s own defense system. Plants activate their immune system in response to fungi, but the plant could suffer from this strong immune response, as is the case in allergic reactions to exogenous molecules. Unfortunately, our results do not differentiate between these hypotheses, but this may be an interesting question for future investigation.

In this study, we found that rust disease entailed metabolic deficiency in the ANM pathway in Rosaceae hosts. This deficiency arises due to abnormal sugar alcohol accumulation induced by *Gymnosporangium*. This novel relationship between metabolite accumulation and deficiency was identified using an optimized statistical model, which ultimately showed the interaction effects between parasitism and metabolic deficiency in the ANM pathway. As a result, phytopathologic clues about host responses were identified, reflecting the intrinsic causal agent in a metabolic network. These findings suggest a new approach for understanding the complex phytopathology of rust disease, focusing on the metabolic response of the host. Additionally, given that products of the ANM pathway are closely related to cell wall biosynthesis, these results provide a new interpretation of this pathology, thus enabling advances in agriculture and plant science.

## Methods

### Plant sampling

Control and parasitized leaves (in the aecial stage) from *Crataegus pinnatifida*, *Chaenomeles sinensis* and *Pyrus pyrifolia* (n = 10 for both groups; 60 samples in total) were collected from trees at different sites. Leaves, fruits and petioles of every tree exhibited short white cylindrical aecia on swollen host tissue, which is a symptom of aecial stage in rust disease cycle. To control for potential atypical results in metabolite alterations, we collected six additional samples from each species at the same sites (n = 3 for both control and parasitized leaves) one year after collecting the first 60 samples. All samples were immediately placed at −40 °C after collection and were then stored in a −70 °C deep freezer. The leaves were separated into three parts: aecia for fungal species identification; a ‘parasitism’ group with swelling lesions on deformed leaves after detaching the aecium; and a ‘control’ group, with normal leaves without a petiole, midrib or veins. Any remaining fungi on the leaves were removed using distilled water. The leaf samples were then freeze-dried for 24 h, powdered using a grinder (DA700, Daesung Artlon, Korea), and finally passed through sieves (<125 μm) to obtain reproducibly extracted metabolites.

### Fungal identification

Nine aecial specimens of *Gymnosporangium* collected on *Crataegus pinnatifida*, *Chaenomeles sinensis*, and *Pyrus pyrifolia* from Goyang-si, Gyeonggi-do, South Korea. A compound light microscope (Zeiss Axiophot, Germany) was used to observe characteristics of specimens examined, including shape and size of the peridium and peridial cells; shape, size and color of aeciospores; and thickness of the cell walls of aeciospores at a magnification of 400x. Unstained slides were prepared from a piece of aecium using distilled water. Thirty spores of each specimen were observed. Based on morphological characteristics and host range, the rust fungus was determined to be *Gymnosporangium asiaticum.* The aecial specimens were deposited at the Herbarium of Braintree Biotechnology Institute, Seoul, Korea (BBI-HY); HY2924, HY2925, HY2926, HY2927, HY2928, HY2929, HY2930, HY2931, HY2931. All aecial morphological characters closely matched the published description of *Gymnosporangium*^37^.

### Metabolite profiling

Metabolites in ground leaves (20 mg) were extracted by sonication for 30 min in ice-cold 1:2.5:1 chloroform:methanol:water (HPLC grade, J.T. Baker, USA). Heptadecanoic acid, a C17:0 fatty acid (Sigma-Aldrich, USA), was added as an internal standard. After centrifugation at 13,000 × g for 5 min, 500 μL of the supernatant was dried under N_2_ gas. The metabolites were then derivatized for detection by GC-MS. Methoxyamine hydrochloride solution (100 μL of 20 mg mL^−1^ in pyridine, Sigma-Aldrich) was added to the extracted metabolites, and methoxyamination proceeded for 90 min at 37 °C. Subsequently, the same volume of BSTFA solution (N,O-bis(trimethylsilyl)trifluoroacetamide with 1% trimethylchlorosilane, Sigma-Aldrich) was used for trimethylsilylation during derivatization for 25 min at 50 °C^38^,^39^. The derivatized samples were analyzed using a GCMS-2010 plus (Shimadzu, Japan) equipped with a DB-5 capillary column (30 m × 0.25 mm, 0.25 μm thickness, Agilent, USA). Samples (1 μL) were injected at an injection temperature of 300 °C and a 1:10 split ratio. High-purity helium (1 mL min^−1^ constant flow) was used as the carrier gas with the following oven temperature program: an initial hold at 100 °C for 2 min, an increase to 300 °C at 6 °C min^−1^ and a hold at 300 °C for 5 min, for a total of 40.33 min. Separated compounds were ionized under electron impact (EI) mode with a 70 eV filament and an ion source at 250 °C. The detection range was 40 to 600 m/z, with a scan rate of 5,000 s^−1^. The retention index solution (C7-C40 alkane mixture, Sigma-Aldrich) was measured, and metabolites were assigned by comparing the derived retention index and deconvoluted mass spectrum of each peak (Supplementary Fig. S10) with those of metabolite standards using AMDIS equipped with the NIST mass spectral library (NIST08, USA). For several sugars and sugar alcohols that share the same molecular formula but have different OH group stereochemistry, standard data using reference compounds for each (Sigma-Aldrich) were acquired, after which the retention time and mass spectrum of each metabolite were confirmed. We also attempted to quantify glucuronic acid, galacturonic acid and fucose using standard compounds; we checked both the linearity and limit of detection using a procedure identical to that for metabolite profiling but found that these compounds were not present in all leaves.

### Data processing and statistics

The data for all 60 samples were aligned using MZmine (version 2.10, http://mzmine.sourceforge.net/), a useful tool for both LC-MS and GC-MS data processing, after exporting all of the data into a *.cdf file^40^,^41^. Next, mass detection was performed using a centroid, chromatogram building and chromatogram deconvolution with the Savitzky-Golay algorithm and isotopic peak grouping, after which we applied the RANSAC algorithm to align the 60 data points with the following parameters: retention time tolerance of 1; retention time tolerance after correction of 0.5; iterations of 50,000; minimum number of points of 0.2%; and a threshold value of 4. The aligned bucket table in SIMCA-P + (version 12.0, Umetrics, Sweden) was used to acquire principal component analysis (PCA) and partial least square-discriminant analysis (PLS-DA) results. These multivariate statistical analyses adopted centering and scaling to 1/standard deviation as a base weight and normalization against the internal standard area. Unpaired t-tests (p-value under 0.05) and variable importance for projection value (value over 1.00) were used as criteria for marker determination, and fold change was measured on the log scale. Hierarchical clustering analysis based on Pearson’s correlation was conducted with log-transformed and mean-centered data using MetaboAnalyst 3.0 (http://metaboanalyst.ca/)^42^. To check for correlations among the detected metabolites, the Pearson’s product-moment correlation coefficient was measured, yielding values between +1 (positive correlation, blue) and −1 (negative correlation, red). This coefficient was applied both in the correlation heat-maps and for metabolic pathway mapping, which was performed using VANTED (version 2.1.0, http://vanted.ipk-gatersleben.de/) with the SBGN-ED add-on^43^. Metabolic pathways [fructose and mannose metabolism (FMM), pentose and glucuronate interconversion (PGI), galactose metabolism (GM) and amino sugar and nucleotide sugar metabolism (ANM)] were downloaded in the KGML format for metabolic pathway mapping, and enzymes that are intermediates of the integrated PPP (Supplementary Table S4) were researched in Kyoto Encyclopedia of Genes and Genomes (KEGG, http://www.genome.jp/kegg/). For pathway enrichment, we used MBrole (http://csbg.cnb.csic.es/mbrole/) with the KEGG pathway database^44^. Scatter column plots using log-scaled data were derived from GraphPad Prism (version 5, GraphPad Software, USA), and the radar chart was generated using Excel (version 2013, Microsoft, USA). Interaction effects between 2 continuous variables (accumulated metabolites and ANM supplies) and 1 nominal variable (control/parasitism) were measured using SPSS (version 22, IBM, New York, USA) and R software (version 3.2.2).

## Additional Information

**How to cite this article**: Lee, D.-K. *et al.* Metabolic response induced by parasitic plant-fungus interactions hinder amino sugar and nucleotide sugar metabolism in the host. *Sci. Rep.* **6**, 37434; doi: 10.1038/srep37434 (2016).

**Publisher’s note:** Springer Nature remains neutral with regard to jurisdictional claims in published maps and institutional affiliations.

## Supplementary Material

Supplementary Information

## Figures and Tables

**Table 1:** Direction, adjusted R square and p-values of the interaction effect between sugars (fructose, arabinose and galactose; dependent variables) and sugar alcohols (ribitol, arabitol, erythritol, mannitol and xylitol; independent variables).

Species	Metabolite	Fructose			Arabinose			Galactose		
		Effect direction	Adjusted R square	p-value	Effect direction	Adjusted R square	p-value	Effect direction	Adjusted R square	p-value
CP	Ribitol	+	0.534	0.346	+	0.853	0.184	−	0.584	0.854
	Arabitol	−	0.924	<0.001	−	0.983	<0.001	−	0.787	0.102
	Erythritol	−	0.497	0.421	+	0.837	0.436	−	0.594	0.872
	Mannitol	−	0.888	<0.001	−	0.953	<0.001	−	0.775	0.070
	Xylitol	−	0.914	<0.001	−	0.982	<0.001	−	0.760	0.020
PS	Ribitol	+	0.577	0.497	−	0.743	0.277	−	0.797	0.868
	Arabitol	+	0.625	0.039	−	0.567	0.014	−	0.769	0.528
	Erythritol	+	0.520	0.695	+	0.789	0.868	−	0.892	0.637
	Mannitol	−	0.532	0.952	+	0.548	0.743	−	0.769	0.781
	Xylitol	+	0.640	0.032	−	0.888	0.150	−	0.852	0.237
CS	Ribitol	+	0.909	0.980	−	0.816	0.158	+	0.563	0.144
	Arabitol	−	0.967	0.028	−	0.925	0.028	−	0.841	0.120
	Erythritol	−	0.953	0.067	−	0.893	0.002	−	0.771	0.241
	Mannitol	−	0.969	0.012	−	0.866	0.018	−	0.787	0.348
	Xylitol	−	0.981	0.180	−	0.946	0.041	−	0.811	0.003

Adjusted R square indicates a degree of interaction between them, and the p-value indicates the reliability of the effect.

CP: *Crataegus pinnatifida.*

CS: *Chaenomeles sinensis.*

PS: *Pyrus pyrifolia.*

## References

[b1] Mendgen K. & Hahn M. Plant infection and the establishment of fungal biotrophy. Trends Plant Sci. 7, 352–356 (2002). 12167330 10.1016/s1360-1385(02)02297-5

[b2] Spanu P. D. *et al.* Genome Expansion and Gene Loss in Powdery Mildew Fungi Reveal Tradeoffs in Extreme Parasitism. Science 330, 1543–1546 (2010). 21148392 10.1126/science.1194573

[b3] Lyu X. *et al.* Comparative genomic and transcriptional analyses of the carbohydrate-active enzymes and secretomes of phytopathogenic fungi reveal their significant roles during infection and development. Sci. Rep. 5, 15565 10.1038/srep15565 (2015). 26531059 PMC4632110

[b4] Misra R. C., Sandeep, Kamthan M., Kumar S. & Ghosh S. A thaumatin-like protein of Ocimum basilicum confers tolerance to fungal pathogen and abiotic stress in transgenic Arabidopsis. Sci. Rep. 6, 25340 10.1038/srep25340 (2016). 27150014 PMC4858651

[b5] Udvardi M. & Poole P. S. Transport and Metabolism in Legume-Rhizobia Symbioses. Annu. Rev. Plant Biol. 64, 781–805 (2013). 23451778 10.1146/annurev-arplant-050312-120235

[b6] Hofmann J. *et al.* Metabolic profiling reveals local and systemic responses of host plants to nematode parasitism. Plant J. 62, 1058–1071 (2010). 20374527 10.1111/j.1365-313X.2010.04217.x PMC2904900

[b7] Jeelani G. & Nozaki T. Metabolomic analysis of Entamoeba: applications and implications. Curr. Opin. Microbiol. 20, 118–124 (2014). 24950028 10.1016/j.mib.2014.05.016

[b8] Yun H.-Y., Lee S.-K. & Lee K.-J. Identification of Aecial Host Ranges of Four Korean *Gymnosporangium* Species Based on the Artificial Inoculation with Teliospores Obtained from Various Forms of Telia. Plant Pathol. J. 21, 310–316 (2005).

[b9] Harrington T. C., McNew D. & Yun H. Y. Bur oak blight, a new disease on Quercus macrocarpa caused by Tubakia iowensis sp. nov. Mycologia 104, 79–92 (2012). 21937728 10.3852/11-112

[b10] Van der Auwera G., Chapelle S. & De Wächter R. Structure of the large ribosomal subunit RNA of Phytophthora megasperma, and phylogeny of the oomycetes. FEBS Lett. 338, 133–136 (1994). 8307170 10.1016/0014-5793(94)80350-1

[b11] Yun H. Y. *et al.* The rust fungus Gymnosporangium in Korea including two new species, G. monticola and G. unicorne. Mycologia 101, 790–809 (2009). 19927745 10.3852/08-221

[b12] Jeon Y.-S. *et al.* jPHYDIT: a JAVA-based integrated environment for molecular phylogeny of ribosomal RNA sequences. Bioinformatics 21, 3171–3173 (2005). 15855247 10.1093/bioinformatics/bti463

[b13] Eriksson L., Trygg J. & Wold S. CV-ANOVA for significance testing of PLS and OPLS^®^ models. J. Chemometr. 22, 594–600 (2008).

[b14] Bowne J. B. *et al.* Drought Responses of Leaf Tissues from Wheat Cultivars of Differing Drought Tolerance at the Metabolite Level. Mol. Plant 5, 418–429 (2012). 22207720 10.1093/mp/ssr114

[b15] Huck J. H. J., Struys E. A., Verhoeven N. M., Jakobs C. & van der Knaap M. S. Profiling of Pentose Phosphate Pathway Intermediates in Blood Spots by Tandem Mass Spectrometry: Application to Transaldolase Deficiency. Clin. Chem. 49, 1375–1380 (2003). 12881455 10.1373/49.8.1375

[b16] Solomon P. S., Waters O. D. & Oliver R. P. Decoding the mannitol enigma in filamentous fungi. Trends Microbiol. 15, 257–262 (2007). 17442575 10.1016/j.tim.2007.04.002

[b17] Najjar V. A. & Pullman M. E. The Occurrence of a Group Transfer Involving Enzyme (phosphoglucomutase) and Substrate. Science 119, 631–634 (1954). 13156640 10.1126/science.119.3097.631

[b18] Holden H. M., Rayment I. & Thoden J. B. Structure and Function of Enzymes of the Leloir Pathway for Galactose Metabolism. J. Biol. Chem. 278, 43885–43888 (2003). 12923184 10.1074/jbc.R300025200

[b19] Voegele R. T. *et al.* Possible roles for mannitol and mannitol dehydrogenase in the biotrophic plant pathogen *Uromyces fabae*. Plant Physiol. 137, 190–198 (2005). 15618426 10.1104/pp.104.051839 PMC548850

[b20] Link T. *et al.* Characterization of a novel NADP(+)-dependent D-arabitol dehydrogenase from the plant pathogen *Uromyces fabae*. Biochem. J. 389, 289–295 (2005). 15796718 10.1042/BJ20050301 PMC1175105

[b21] Maclean D. J. & Scott K. J. Identification of Glucitol (Sorbitol) and Ribitol in a Rust Fungus*, Puccinia graminis* f. sp. tritici. J. Biol. Chem. 97, 83–89 (1976). 10.1099/00221287-97-1-83 993788

[b22] Pfyffer G. E. *et al.* A further report on the occurrence of acyclic sugar alcohols in fungi. Mycol. Res. 94, 219–222 (1990).

[b23] Schulze-Lefert P. & Panstruga R. Establishment of biotrophy by parasitic fungi and reprogramming of host cells for disease resistance. Annu. Rev. Phytopathol. 41, 641–667 (2003). 14527335 10.1146/annurev.phyto.41.061002.083300

[b24] Bonfante P. & Genre A. Mechanisms underlying beneficial plant–fungus interactions in mycorrhizal symbiosis. Nat. Comm. 1, 48 10.1038/ncomms1046 (2010). 20975705

[b25] Hahn M. & Mendgen K. Signal and nutrient exchange at biotrophic plant–fungus interfaces. Curr. Opin. Plant Biol. 4, 322–327 (2001). 11418342 10.1016/s1369-5266(00)00180-1

[b26] Voegele R. T., Struck C., Hahn M. & Mendgen K. The role of haustoria in sugar supply during infection of broad bean by the rust fungus Uromyces fabae. Proc. Natl. Acad. Sci. USA 98, 8133–8138 (2001). 11390980 10.1073/pnas.131186798 PMC35480

[b27] Clay N. K., Adio A. M., Denoux C., Jander G. & Ausubel F. M. Glucosinolate Metabolites Required for an Arabidopsis Innate Immune Response. Science 323, 95–101 (2009). 19095898 10.1126/science.1164627 PMC2630859

[b28] Luna E. *et al.* Callose Deposition: A Multifaceted Plant Defense Response. Mol. Plant-Microbe Interact. 24, 183–193 (2010). 10.1094/MPMI-07-10-0149 20955078

[b29] Schlupmann H., Bacic A. & Read S. M. Uridine Diphosphate Glucose Metabolism and Callose Synthesis in Cultured Pollen Tubes of *Nicotiana alata* Link et Otto. Plant Physiol. 105, 659–670 (1994). 12232233 10.1104/pp.105.2.659 PMC159407

[b30] Wang Q. *et al.* Identification of a UDP-glucose pyrophosphorylase from cotton (Gossypium hirsutum L.) involved in cellulose biosynthesis in *Arabidopsis thaliana*. Plant Cell Reports 30, 1303–1312 (2011). 21373794 10.1007/s00299-011-1042-x

[b31] Lerouxel O., Cavalier D. M., Liepman A. H. & Keegstra K. Biosynthesis of plant cell wall polysaccharides — a complex process. Curr. Opin. Plant Biol. 9, 621–630 (2006). 17011813 10.1016/j.pbi.2006.09.009

[b32] Wolf S., Hematy K. & Hofte H. Growth control and cell wall signaling in plants. Annu. Rev. Plant Biol. 63, 381–407 (2012). 22224451 10.1146/annurev-arplant-042811-105449

[b33] Cosgrove D. J. Growth of the plant cell wall. Nat. Rev. Mol. Cell Biol. 6, 850–861 (2005). 16261190 10.1038/nrm1746

[b34] Josè-Estanyol M. & Puigdomènech P. Plant cell wall glycoproteins and their genes. Plant Physiol. Biochem. 38, 97–108 (2000).

[b35] Burton R. A., Gidley M. J. & Fincher G. B. Heterogeneity in the chemistry, structure and function of plant cell walls. Nat. Chem. Biol. 6, 724–732 (2010). 20852610 10.1038/nchembio.439

[b36] Olszewski K. L. *et al.* Host-Parasite Interactions Revealed by Plasmodium falciparum Metabolomics. Cell Host Microbe 5, 191–199. 19218089 10.1016/j.chom.2009.01.004 PMC2737466

[b37] Hiratsuka N. The rust flora of Japan. (Tsukuba Shuppankai, 1992).

[b38] Zhou K. *et al.* Metabonomics reveals metabolite changes in biliary atresia infants. J. Proteome Res. 14, 2569–2574 (2015). 25899098 10.1021/acs.jproteome.5b00125 PMC6088235

[b39] Zhao Y. *et al.* A metabolomics study delineating geographical location-associated primary metabolic changes in the leaves of growing tobacco plants by GC-MS and CE-MS. Sci. Rep. 5, 16346 10.1038/srep16346 (2015). 26549189 PMC4637841

[b40] Kind T., Tolstikov V., Fiehn O. & Weiss R. H. A comprehensive urinary metabolomic approach for identifying kidney cancer. Anal. Biochem. 363, 185–195 (2007). 17316536 10.1016/j.ab.2007.01.028

[b41] Katajamaa M. & Orešič M. Data processing for mass spectrometry-based metabolomics. J. Chromatogr. A 1158, 318–328 (2007). 17466315 10.1016/j.chroma.2007.04.021

[b42] Sreekumar A. *et al.* Metabolomic profiles delineate potential role for sarcosine in prostate cancer progression. Nature 457, 910–914 (2009). 19212411 10.1038/nature07762 PMC2724746

[b43] Czauderna T., Klukas C. & Schreiber F. Editing, validating and translating of SBGN maps. Bioinformatics 26, 2340–2341 (2010). 20628075 10.1093/bioinformatics/btq407 PMC2935428

[b44] Chagoyen M. & Pazos F. MBRole: enrichment analysis of metabolomic data. Bioinformatics 27, 730–731 (2011). 21208985 10.1093/bioinformatics/btr001

