# Supplementary material for: Metabolic response induced by parasitic plant-fungus interactions hinder amino sugar and nucleotide sugar metabolism in the host
Source: Sci Rep. 2016 Nov 28;6:37434. doi: 10.1038/srep37434 (PMC5124995; doi:10.1038/srep37434)
Supplement: Supplementary Information [file srep37434-s1.pdf]

Supplementary information

Metabolic response induced by parasitic plant-fungus interactions hinder amino sugar and nucleotide sugar metabolism in the host

Dong-Kyu Lee, Soohyun Ahn, Hae Yoon Cho, Hye Young Yun, Jeong Hill Park, Johan Lim, Jeongmi Lee, Sung Won Kwon

Supplementary Figure S1. Leaf samples of C*rataegus pinnatifida* (control, a; parasitized, b), *Chaenomeles sinensis* (control, c; parasitized, d) and *Pyrus pyrifolia* (control, e; parasitized, f).

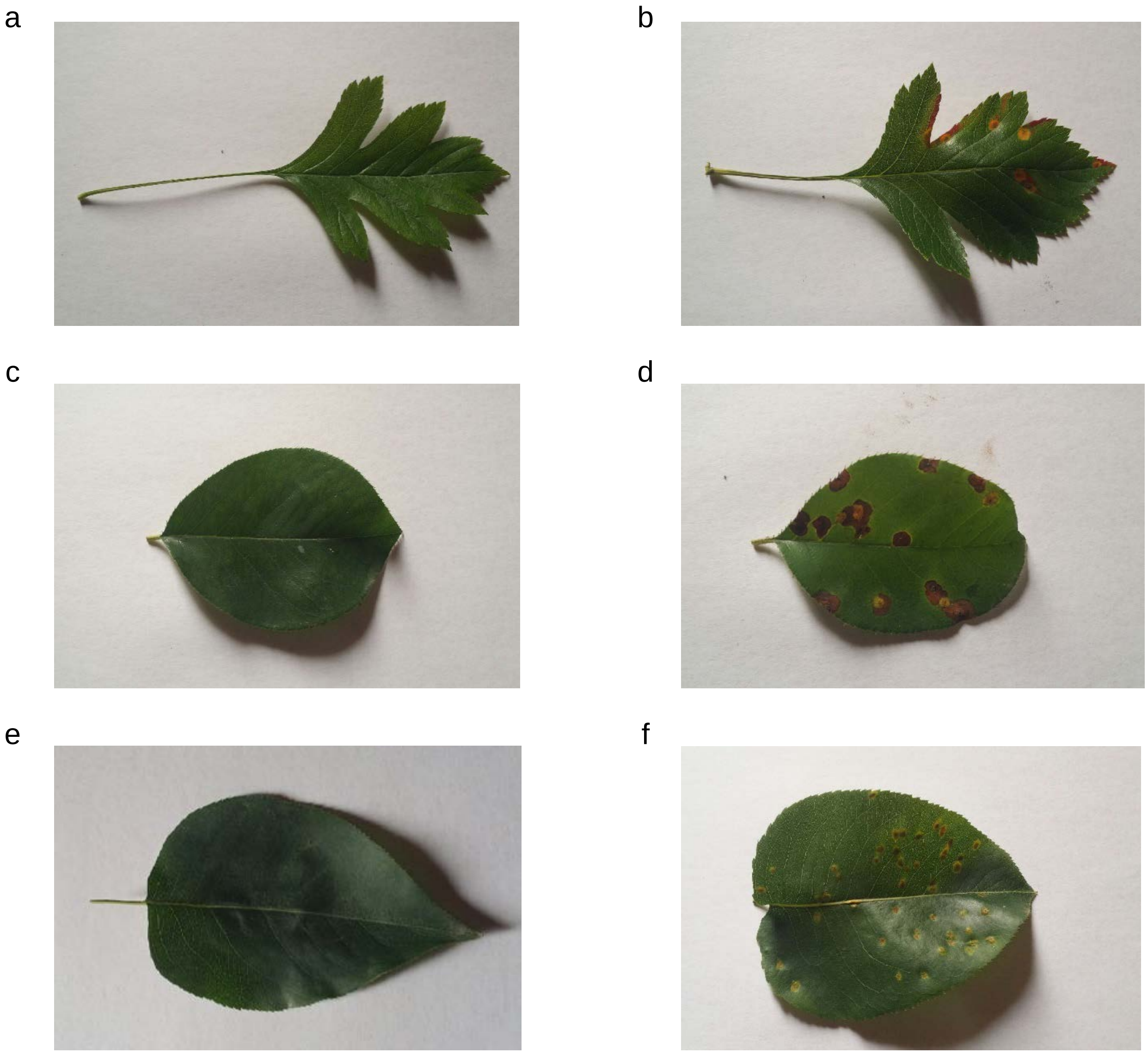

Supplementary Figure S2. Four aeciospores and a single peridial cell of Gymnosporangium asiaticum on Pyrus pyrifolia var. culta (Makino) Nakai, specimen HY2926 (×400).

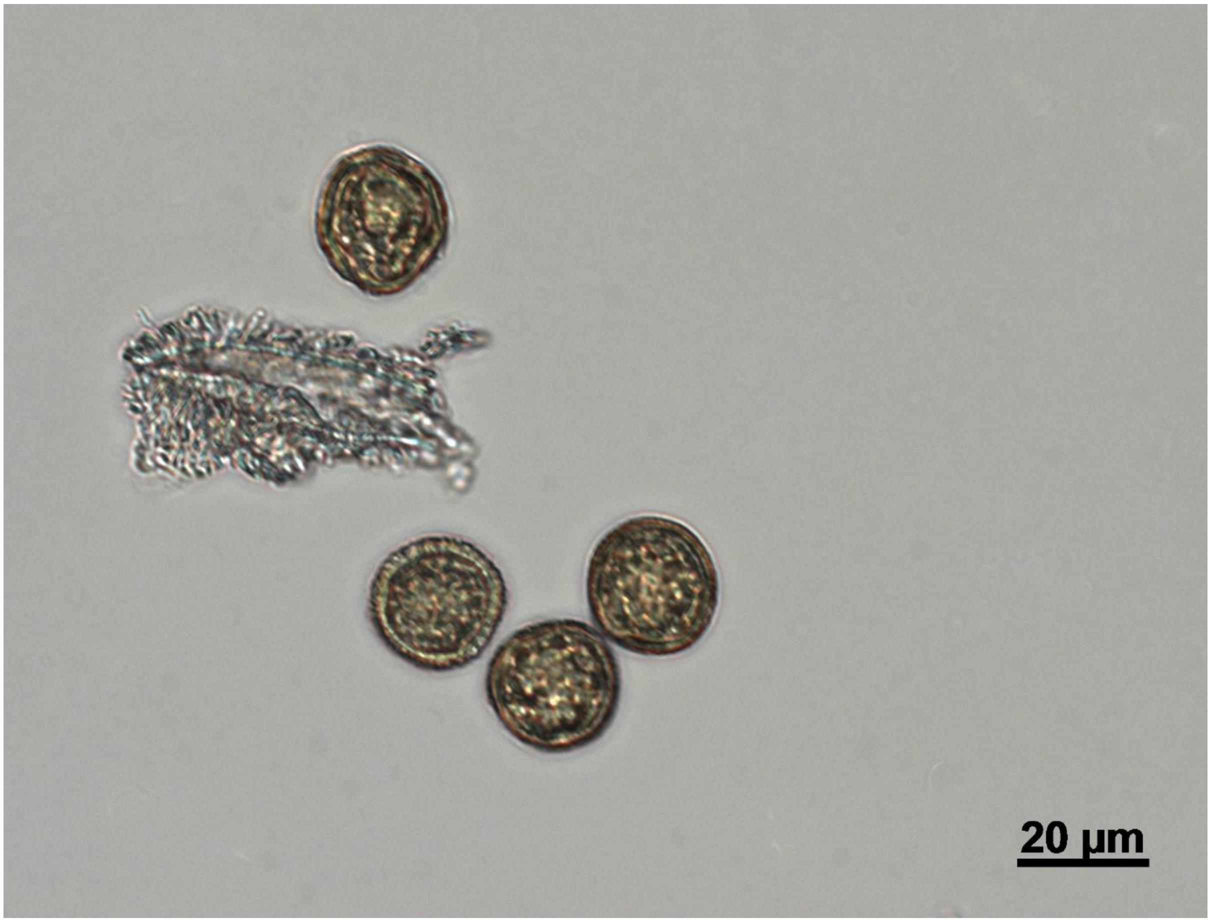

Supplementary Figure S3. PCA score scatter plots of C*rataegus pinnatifida* (n=20), *Chaenomeles sinensis* (n=20) and *Pyrus pyrifolia* (n=20) with control (green) and parasitized (red) groups.

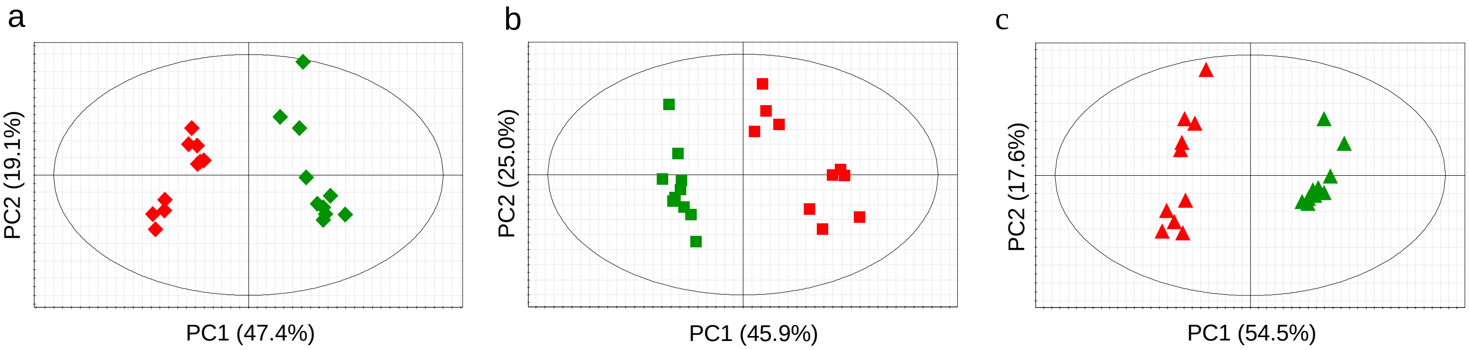

Supplementary Figure S4. PCA score scatter plot of control (green) and parasitized (red), including whole samples of Rosaceae species (n=10 for each group, total n=60)

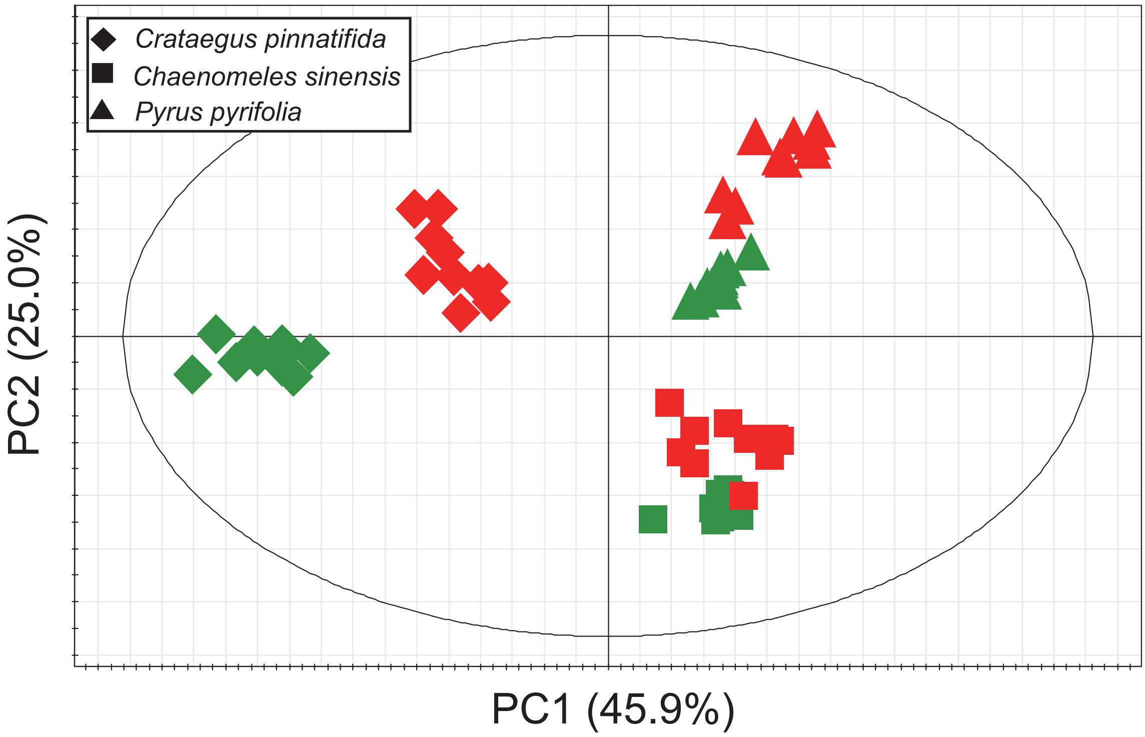

Supplementary Figure S5. Heat-map representation of unsupervised hierachical clustering analysis (HCA)

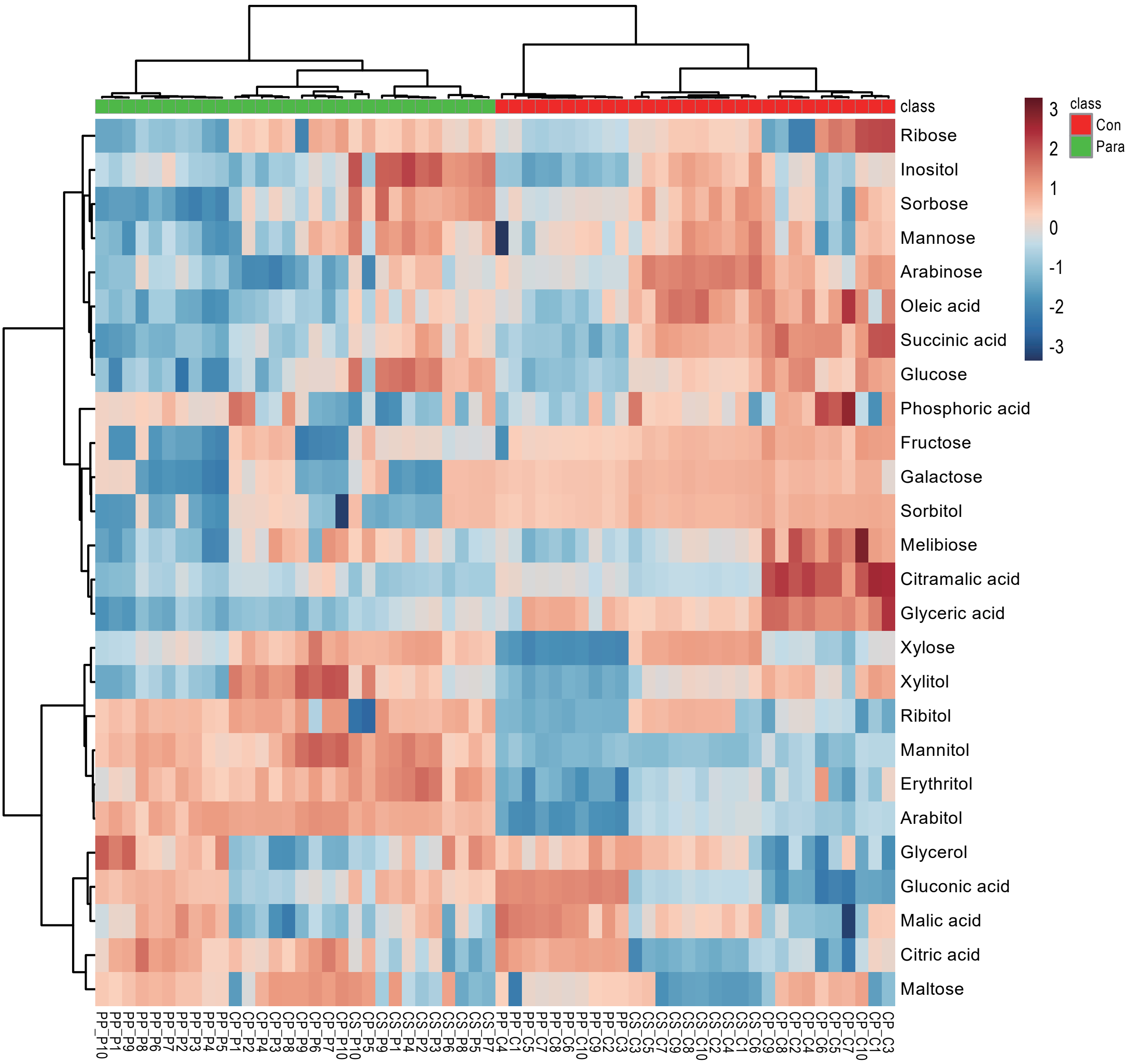

Supplementary Figure S6. Substrates of ANM pathway from other metabolic pathways

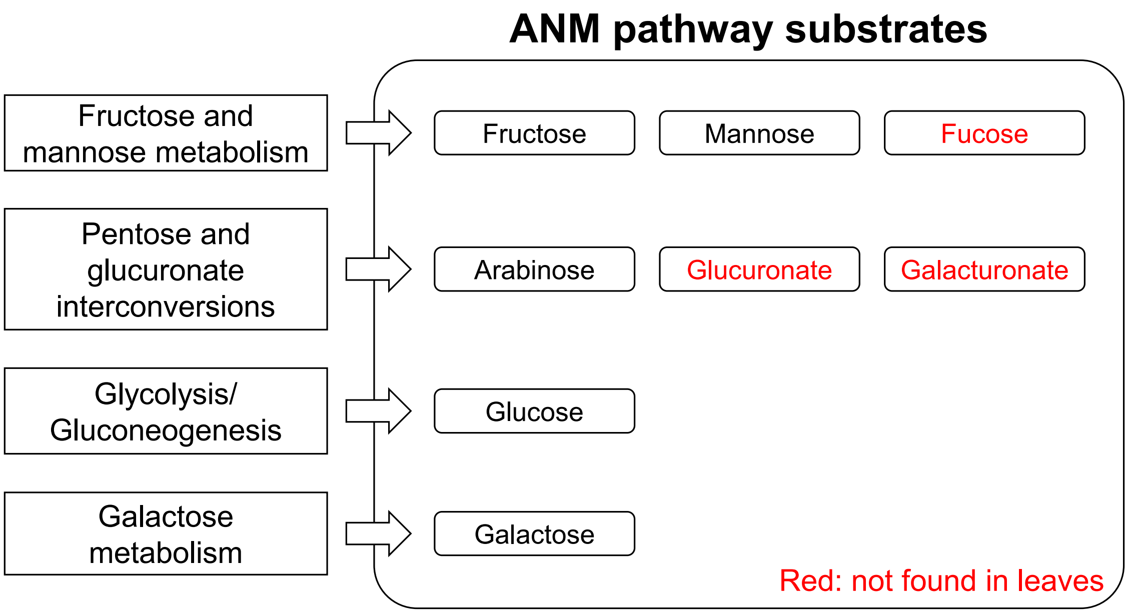

Supplementary Figure S7. Mapping of metabolic alterations on GM. Metabolites (p-value <0.05 as asterisk) between control (green) and parasitism (red) were colored based on PPMCC value from -1 (negative correlation, red) to +1 (positive correlation, blue) against fructose (a), glucose (b) and mannose (c). All values in bar graphs were mean + s.d., 30 replicates for each group.

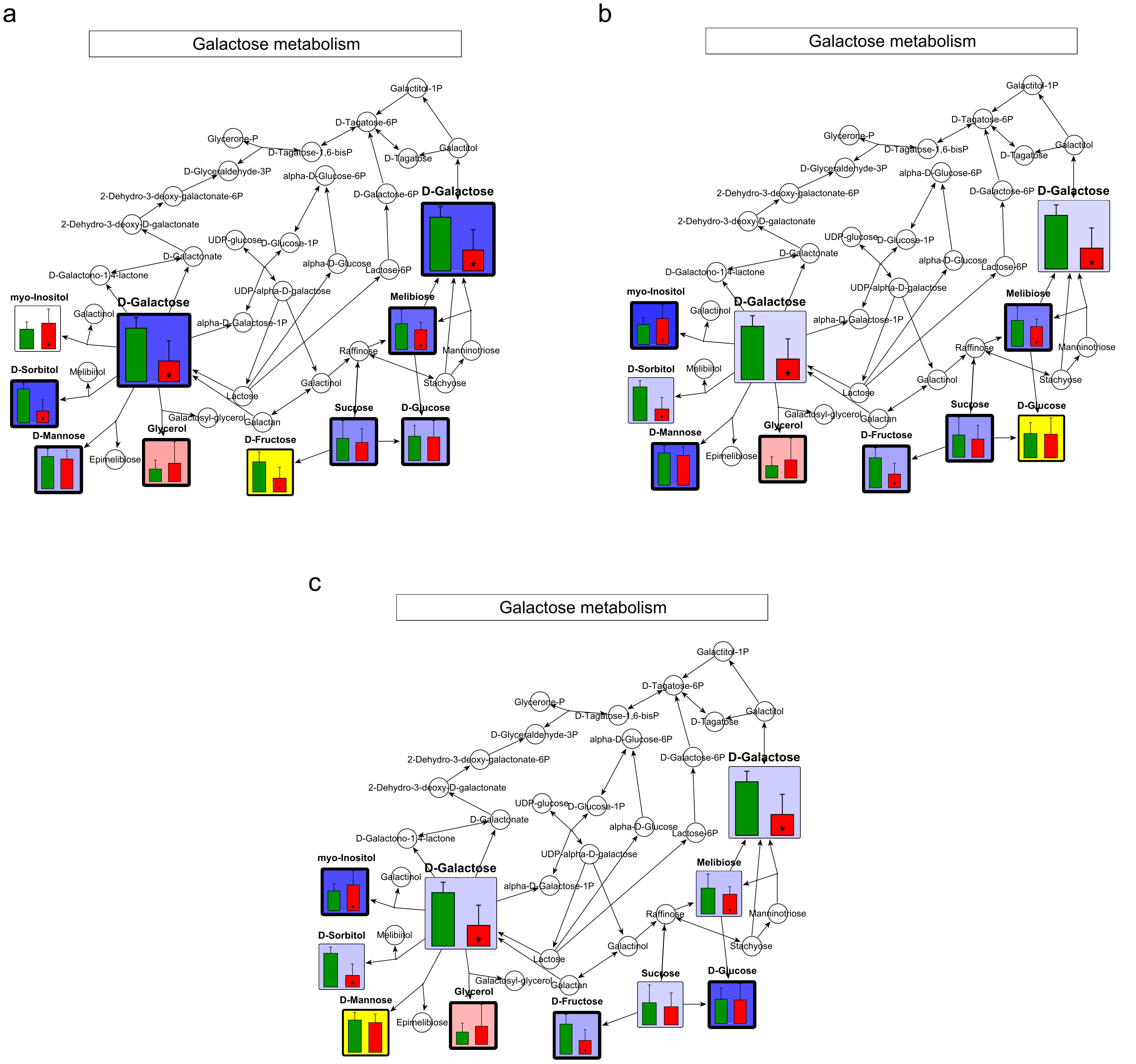

Supplementary Figure S8. Correlation analysis (based on PPMCC) results among whole metabolic changes in *Crataegus pinnatifida* (a, n=20), *Chaenomeles sinensis* (b, n=20) and *Pyrus pyrifolia* (c, n=20). Negative correlations (maximum -1, red) and positive correlations (maximum +1, blue) were displayed on heatmaps with clustering.

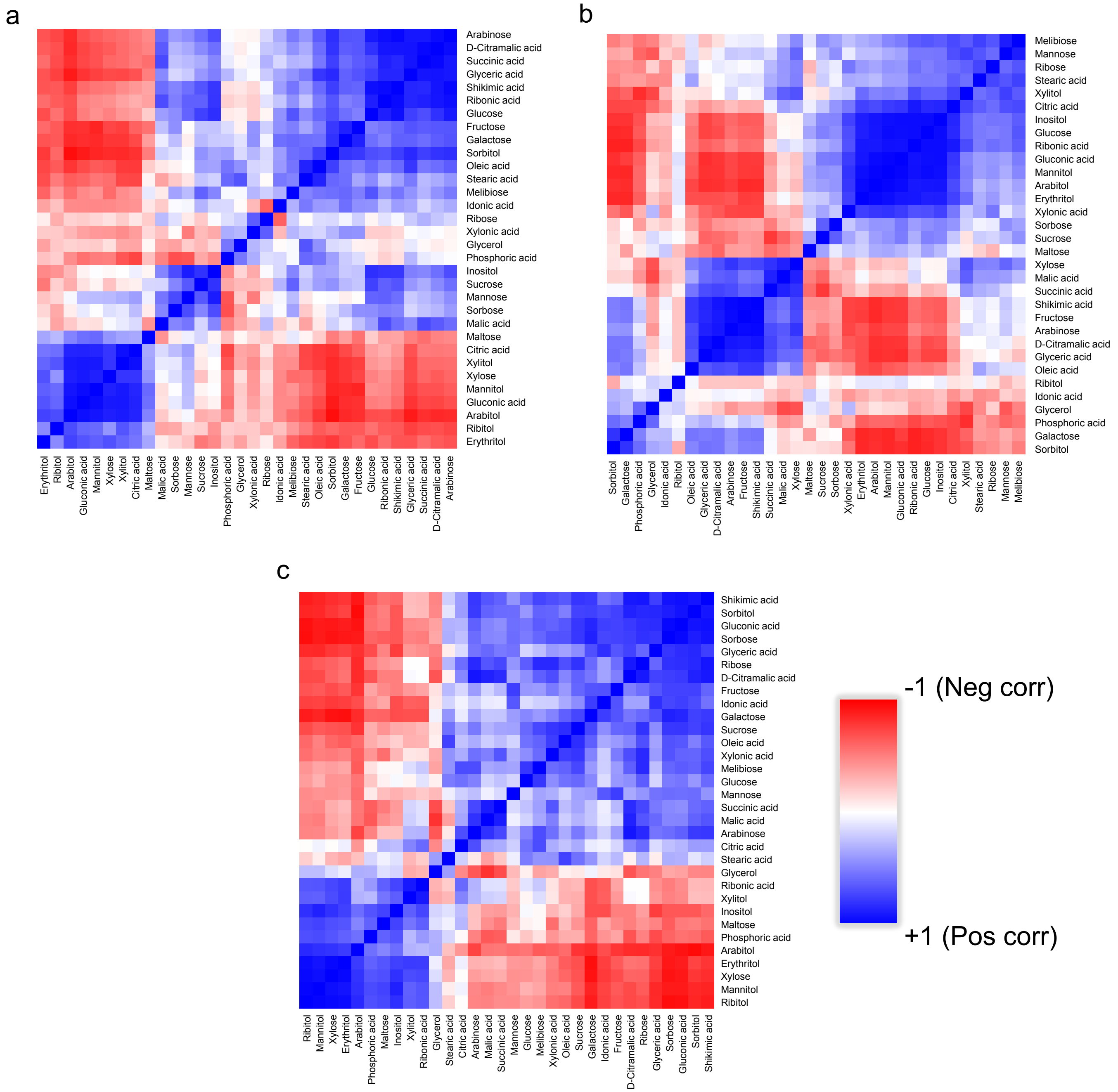

Supplementary Figure S9. Interaction effect of group variable and accumulated sugar alcohols on the ANM supplies. Forty-five scatter plots (n = 15 for each species) with min-max normalized values of ANM supplies (fructose, arabinose and galactose on the y-axis) and accumulated sugar alcohols (ribitol, arabitol, erythritol, mannitol and xylitol on the x-axis). The interaction effect directions of the models were measured by the slope differences of two regression lines upon each group variable (control, green; parasitism, red). The significant models were denoted as *p < 0.05 and **p < 0.01.

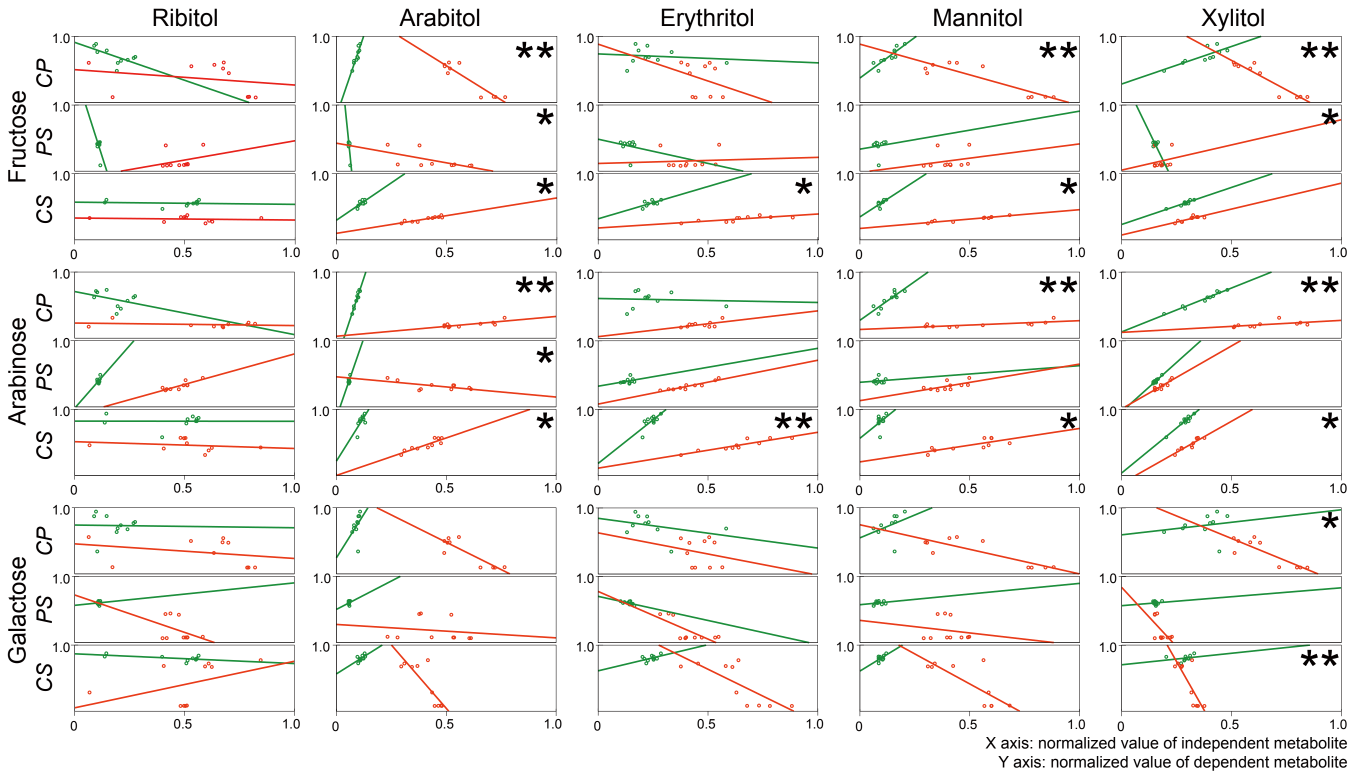

Supplementary Figure S10. Mass spectra of derivatized metabolites. The list of metabolites were as follows: phosphoric acid (a), glycerol (b), succinic acid (c), glyceric acid (d), fumaric acid (e), serine (f), threonine (g), citramalic acid (h), malic acid (i), erythritol (j), aspartic acid (k), erythronic acid (l), 3-hydroxy-3-methylpentanedioic acid (m), xylonic acid (n), xylose (o), arabinose (p), ribose (q), xylitol (r), arabitol (s), ribitol (t), arabinofuranose (u), ribonic acid (v), idonic acid (w), citric acid (x), sorbose (y), mannose (z), fructose (aa), galactose (ab), mannitol (ac), sorbitol (ad), gluconic acid (ae), inositol (af), glucose (ah), linoleic acid (ah), oleic acid (ai), stearic acid (aj), sucrose (ak), maltose (al) and melibiose (am)

(a)

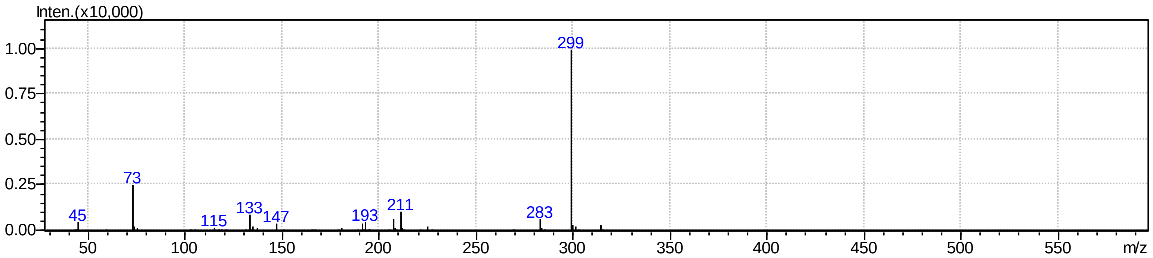

(b)

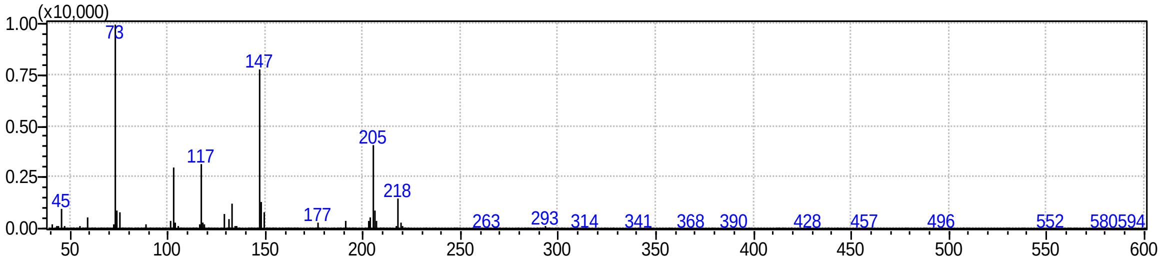

(c)

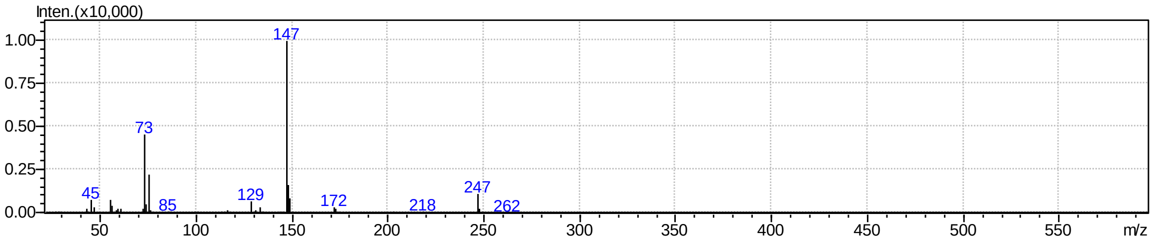

(d)

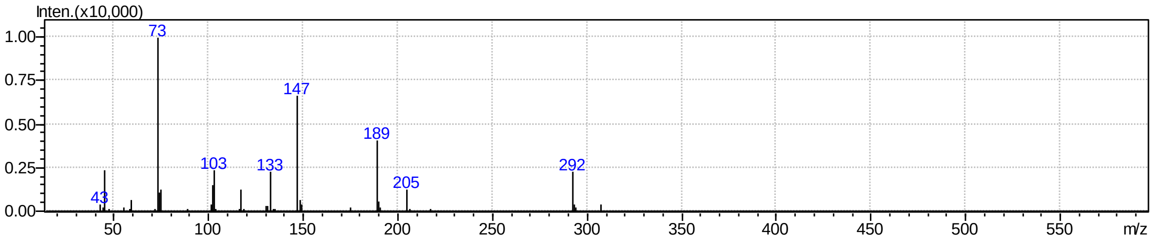

(e)

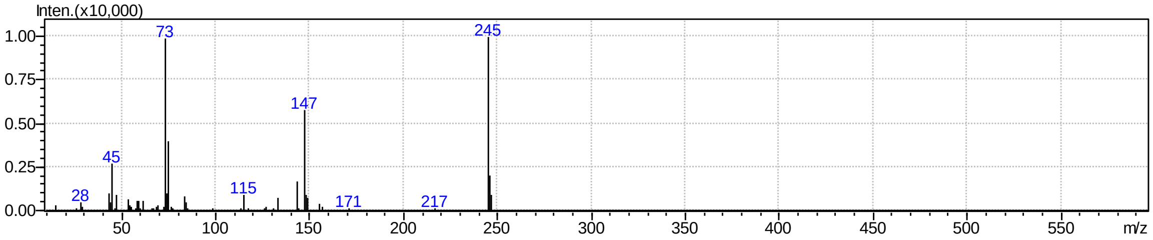

(f)

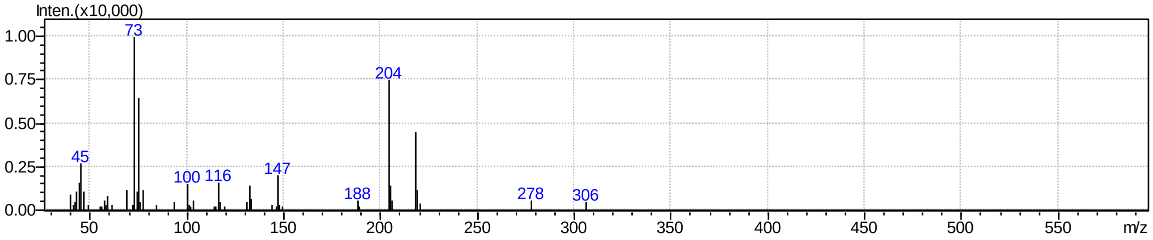
Inten.(x10,000)
1.00 0.75 0.50 0.25 0.00
73
204
45
100 116
147
188
278
306
50 100 150 200 250 300 350 400 450 500 550 m/z

(g)

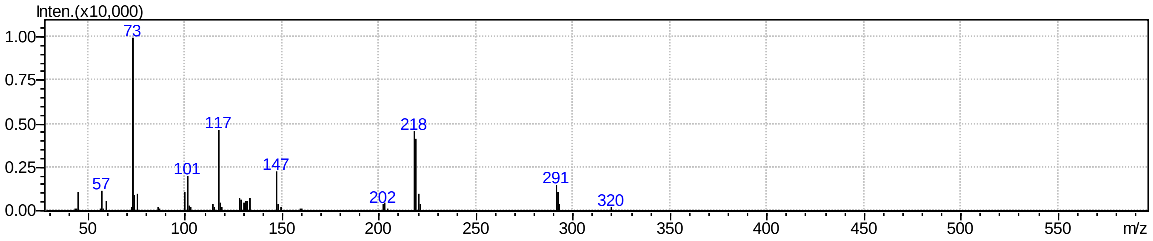
Inten.(x10,000)
1.00 0.75 0.50 0.25 0.00
73
117
218
57
101
147
202
291
320
50 100 150 200 250 300 350 400 450 500 550 m/z

(h)

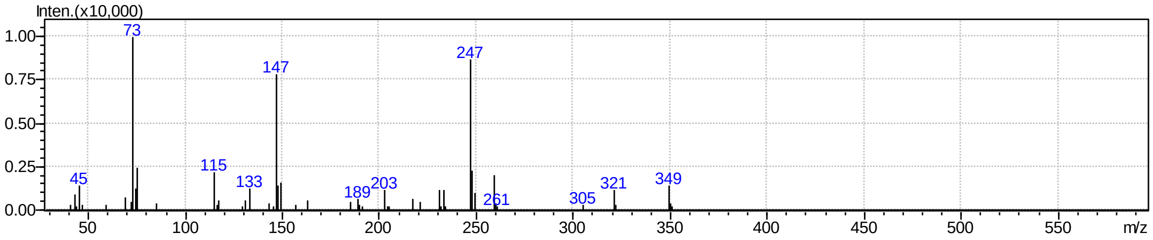
Inten.(x10,000)
1.00 0.75 0.50 0.25 0.00
73
147
247
45
115
133
189 203
261
305
321
349
50 100 150 200 250 300 350 400 450 500 550 m/z

(i)

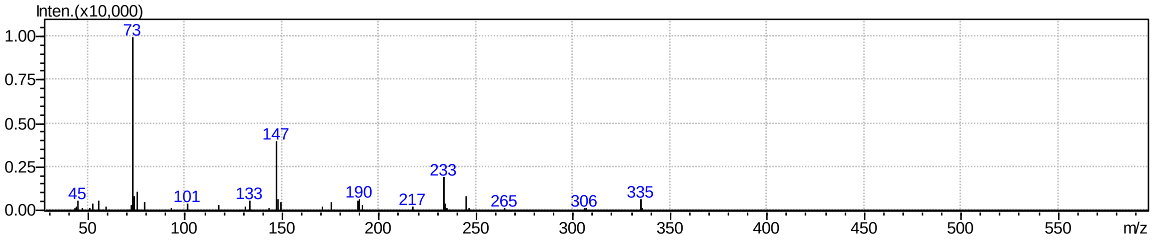
Inten.(x10,000)
1.00 0.75 0.50 0.25 0.00
73
147
233
45
101
133
190
217
265
306
335
50 100 150 200 250 300 350 400 450 500 550 m/z

(j)

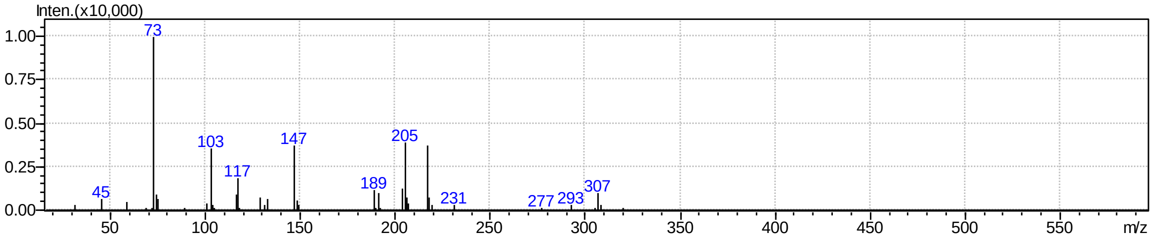
Inten.(x10,000)
1.00 0.75 0.50 0.25 0.00
73
103
147
205
117
45
189
231
277 293
307
50 100 150 200 250 300 350 400 450 500 550 m/z

(k)

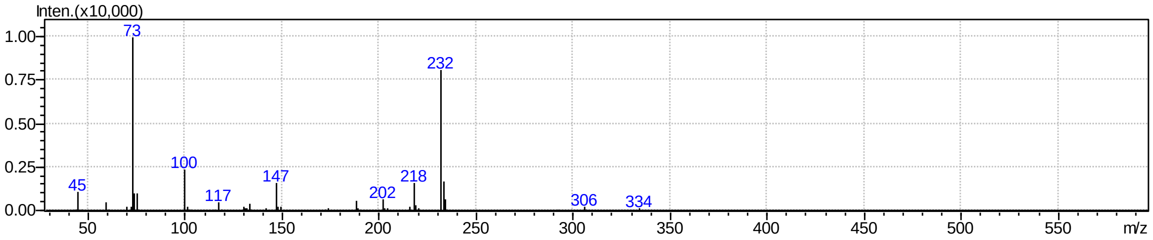
Inten.(x10,000)
1.00 0.75 0.50 0.25 0.00
73
232
45
100
117
147
202
218
306
334
50 100 150 200 250 300 350 400 450 500 550 m/z

(l)

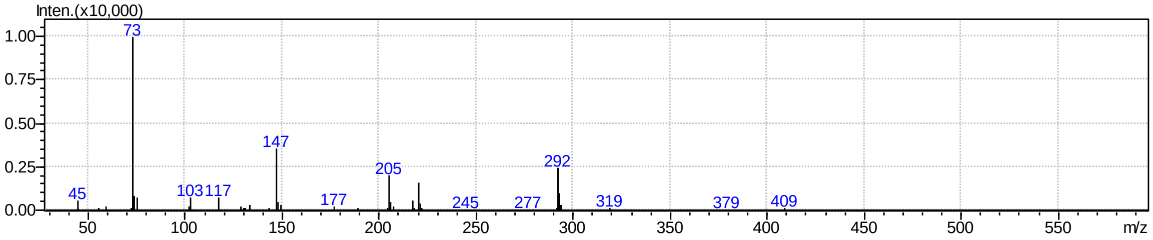
Inten.(x10,000)
73
147
205
292
45
103 117
177
245
277
319
379
409
m/z

(m)

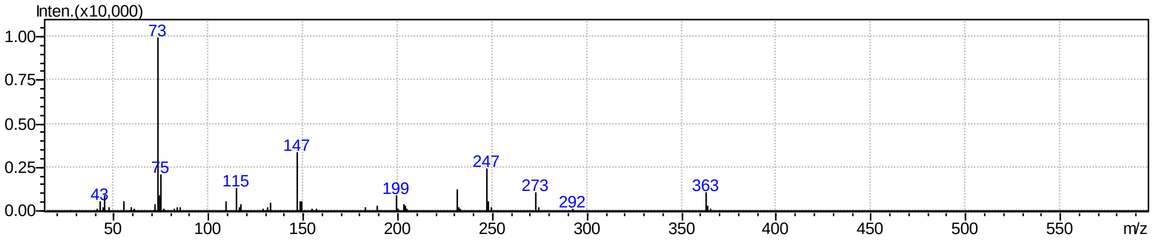
Inten.(x10,000)
73
75
43
115
147
199
247
273
292
363
m/z

(n)

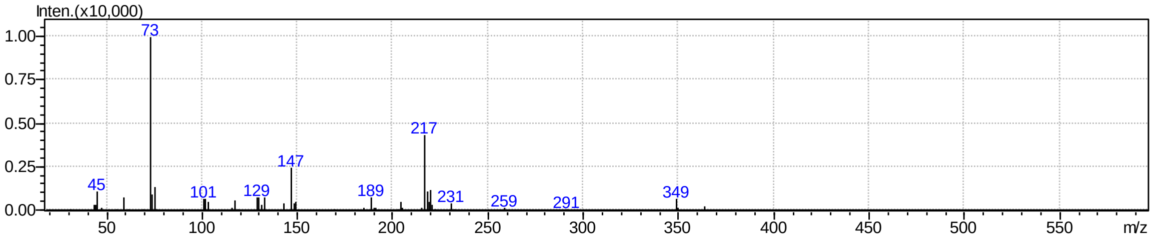
Inten.(x10,000)
73
45
101
129
147
189
217
231
259
291
349
m/z

(o)

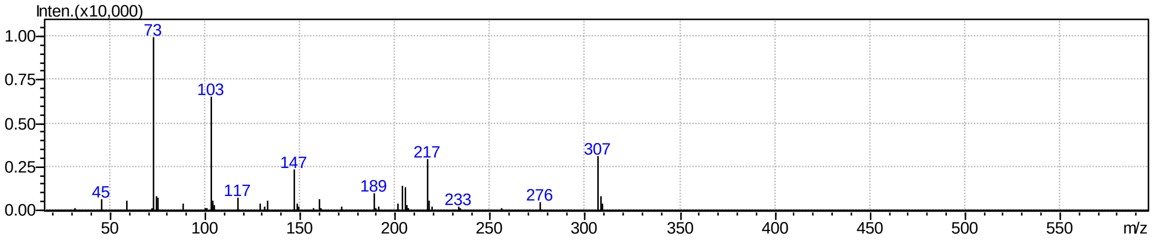
Inten.(x10,000)
73
103
45
117
147
189
217
233
276
307
m/z

(p)

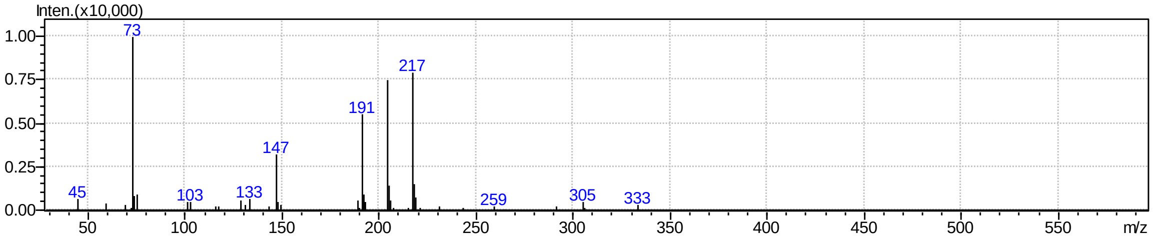
Inten.(x10,000)
73
45
103
133
147
191
217
259
305
333
m/z

(q)

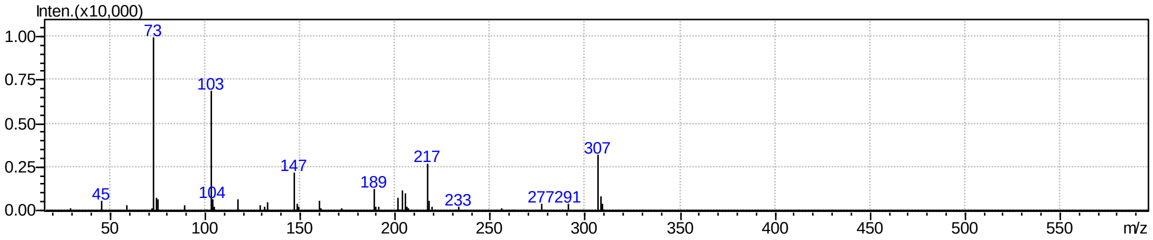
Inten.(x10,000)
73
103
45
104
147
189
217
233
277 291
307
m/z

(r)

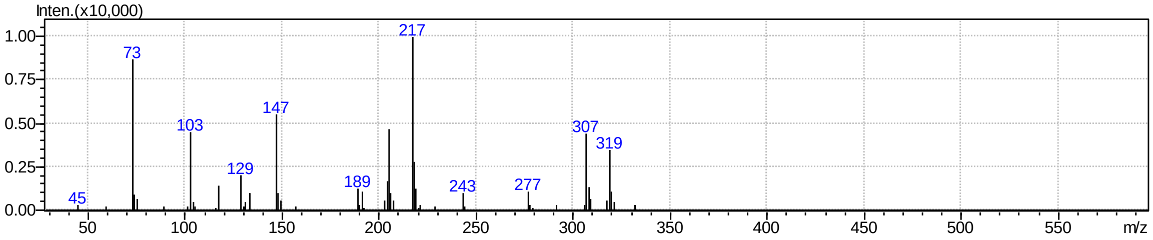
Inten.(x10,000)
1.00
0.75
0.50
0.25
0.00
73
217
103
147
129
189
243
277
307
319
45
50 100 150 200 250 300 350 400 450 500 550 m/z

(s)

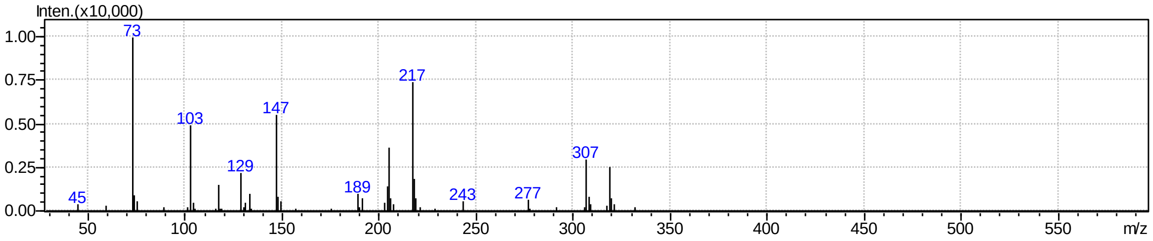
Inten.(x10,000)
1.00
0.75
0.50
0.25
0.00
73
217
103
147
129
189
243
277
307
45
50 100 150 200 250 300 350 400 450 500 550 m/z

(t)

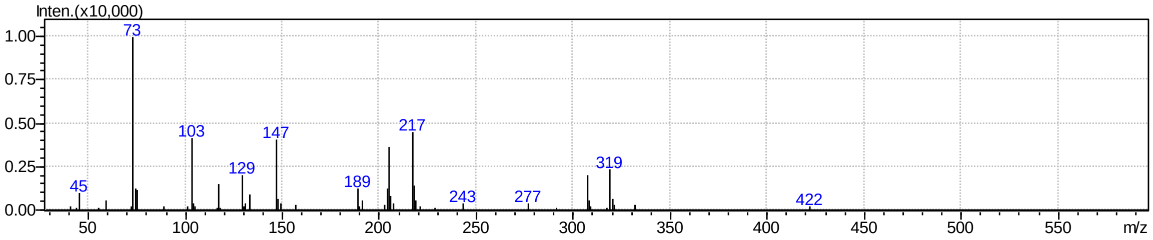
Inten.(x10,000)
1.00
0.75
0.50
0.25
0.00
73
103
147
217
129
189
243
277
319
45
422
50 100 150 200 250 300 350 400 450 500 550 m/z

(u)

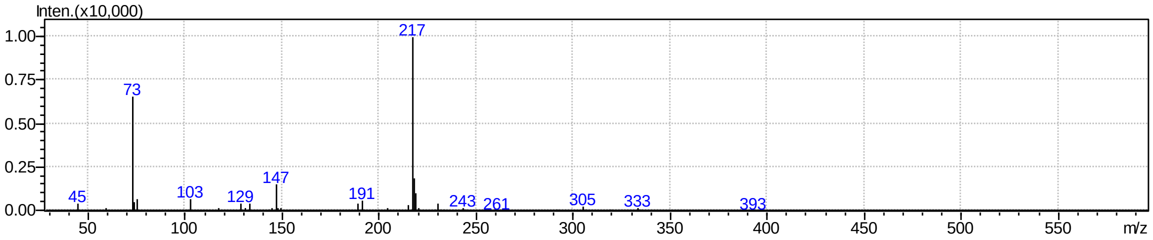
Inten.(x10,000)
1.00
0.75
0.50
0.25
0.00
217
73
45
103
129
147
191
243
261
305
333
393
50 100 150 200 250 300 350 400 450 500 550 m/z

(v)

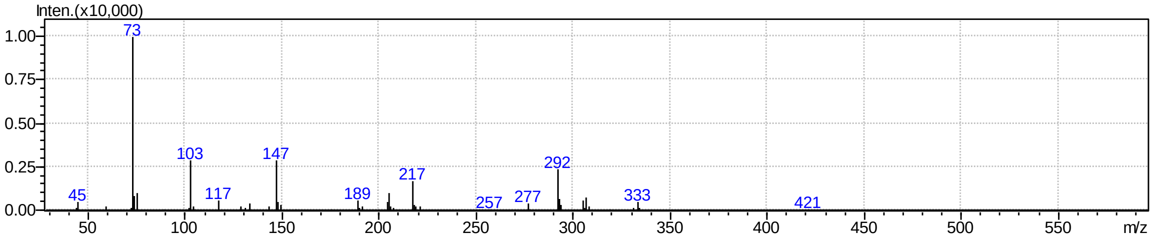
Inten.(x10,000)
1.00
0.75
0.50
0.25
0.00
73
103
147
292
217
45
117
189
257
277
333
421
50 100 150 200 250 300 350 400 450 500 550 m/z

(w)

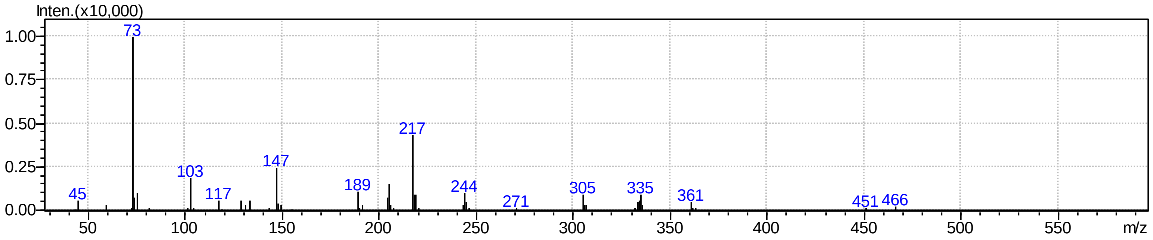
Inten.(x10,000)
1.00
0.75
0.50
0.25
0.00
73
217
147
103
189
244
305
335
45
117
271
361
451
466
50 100 150 200 250 300 350 400 450 500 550 m/z

(x)

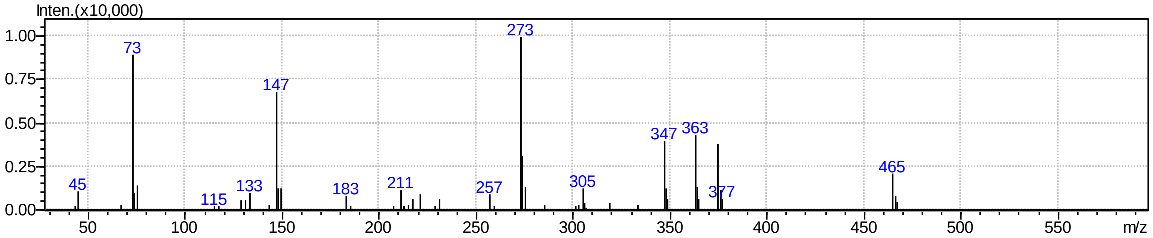
Inten.(x10,000)
1.00 0.75 0.50 0.25 0.00
45 73 115 133 147 183 211 257 273 305 347 363 377 465
50 100 150 200 250 300 350 400 450 500 550 m/z

(y)

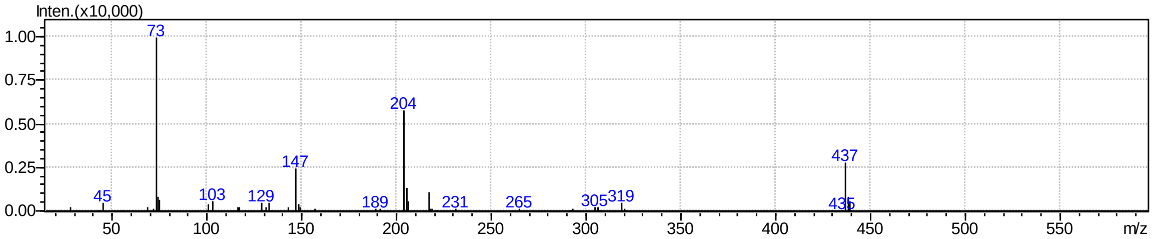
Inten.(x10,000)
1.00 0.75 0.50 0.25 0.00
45 73 103 129 147 189 204 231 265 305 319 435 437
50 100 150 200 250 300 350 400 450 500 550 m/z

(z)

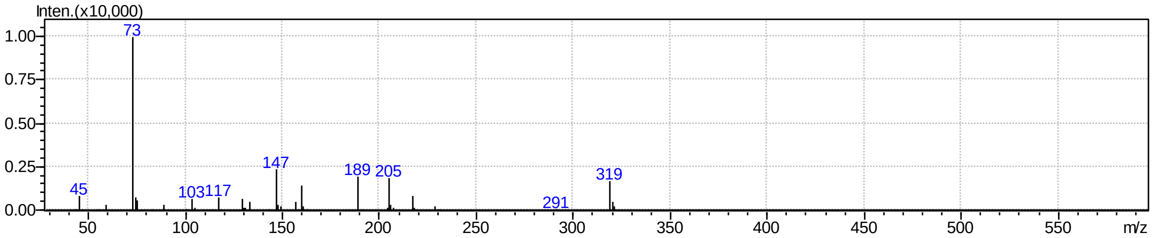
Inten.(x10,000)
1.00 0.75 0.50 0.25 0.00
45 73 103 117 147 189 205 291 319
50 100 150 200 250 300 350 400 450 500 550 m/z

(aa)

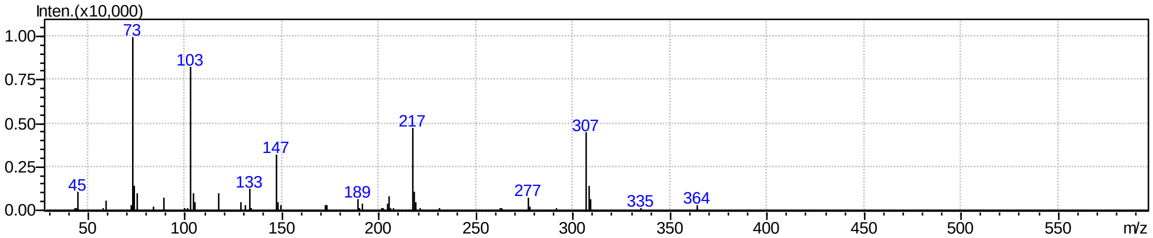
Inten.(x10,000)
1.00 0.75 0.50 0.25 0.00
45 73 103 133 147 189 217 277 307 335 364
50 100 150 200 250 300 350 400 450 500 550 m/z

(ab)

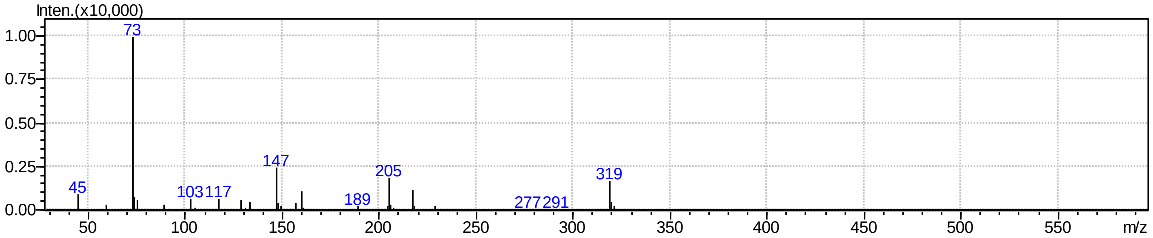
Inten.(x10,000)
1.00 0.75 0.50 0.25 0.00
45 73 103 117 147 189 205 277 291 319
50 100 150 200 250 300 350 400 450 500 550 m/z

(ac)

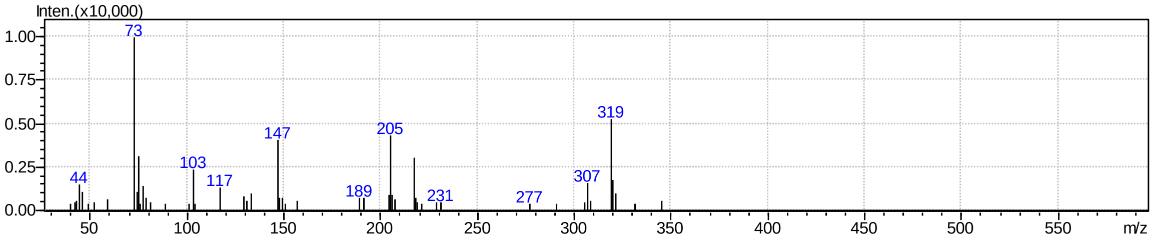
Inten.(x10,000)
1.00 0.75 0.50 0.25 0.00
44 73 103 117 147 189 205 231 277 307 319
50 100 150 200 250 300 350 400 450 500 550 m/z

(ad)

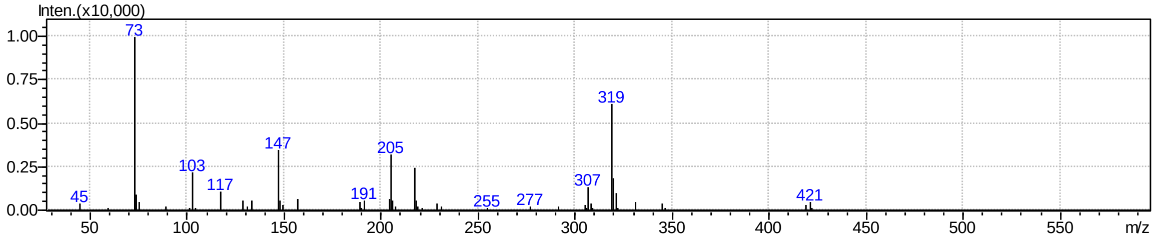

(ae)

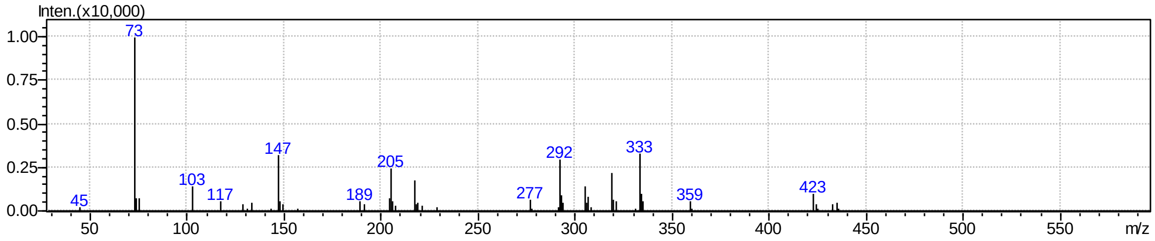

(af)

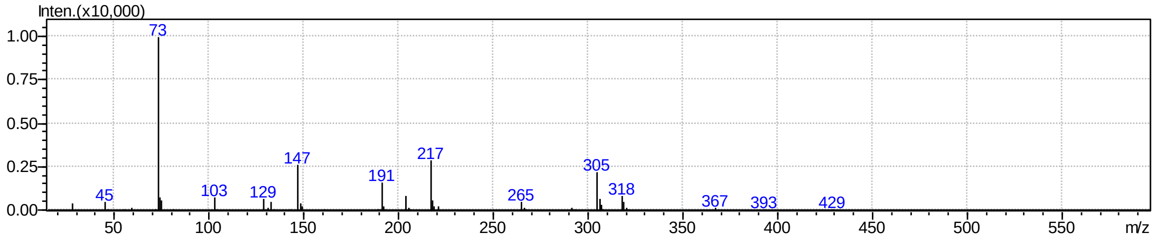

(ag)

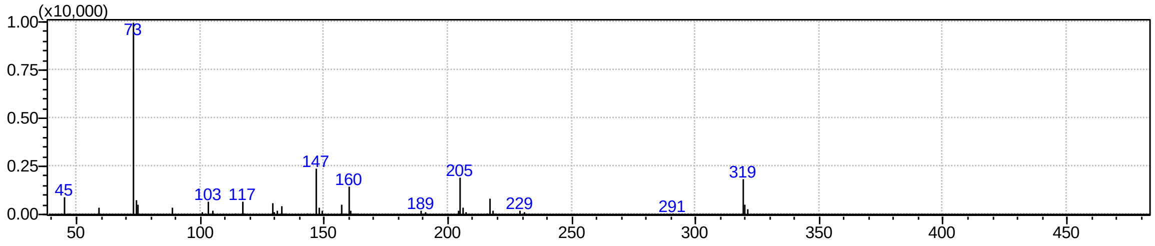

(ah)

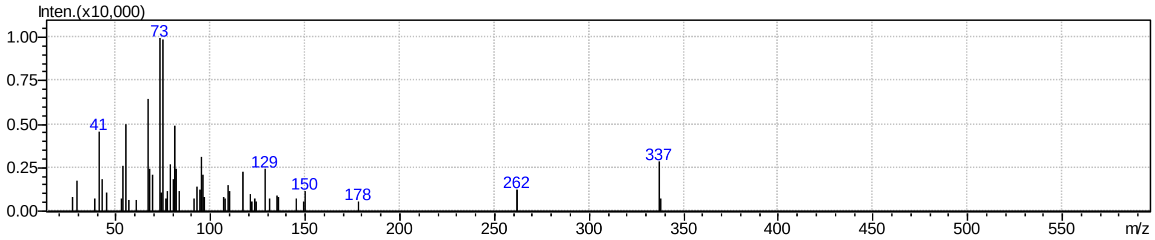

(ai)

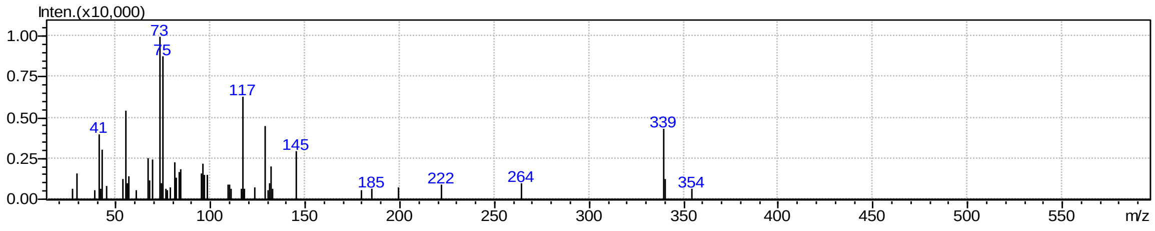

(aj)

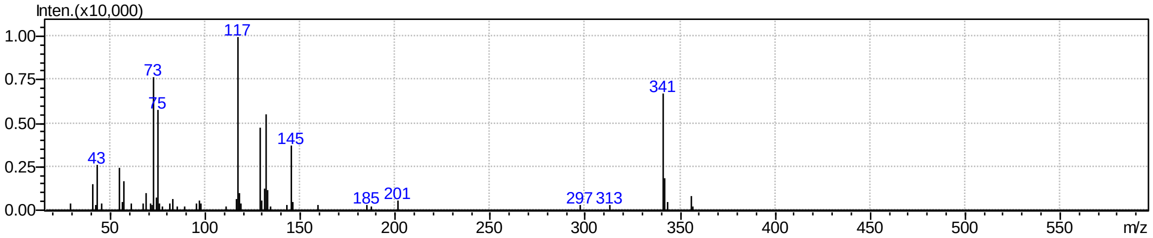
Inten.(x10,000)
117
73
75
341
145
43
185 201
297 313
m/z

(ak)

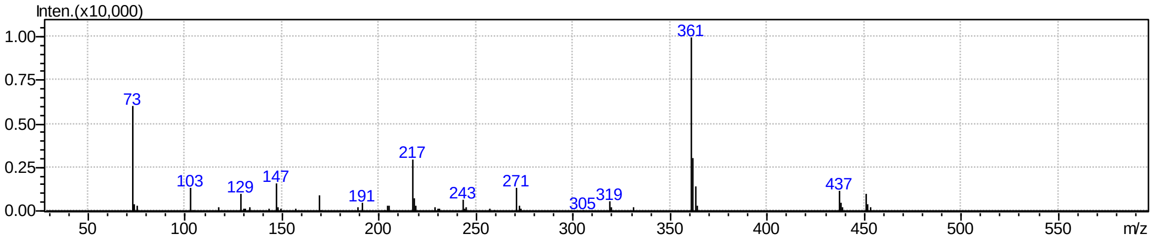
Inten.(x10,000)
361
73
217
103
129
147
191
243
271
305 319
437
m/z

(al)

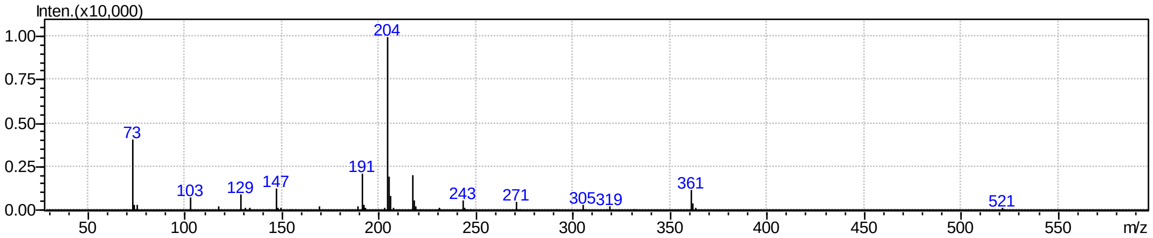
Inten.(x10,000)
204
73
103
129
147
191
243
271
305 319
361
521
m/z

(am)

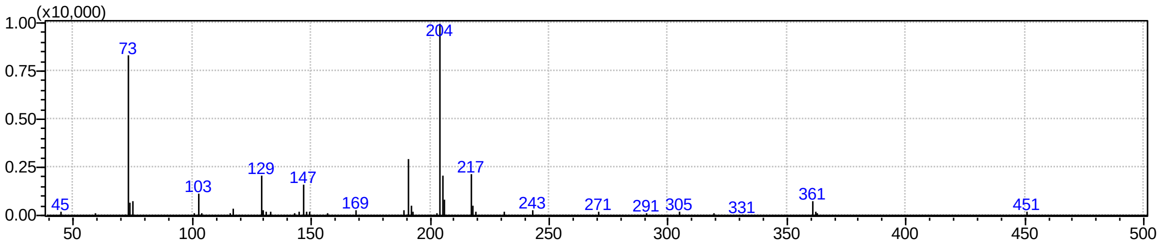
(x10,000)
204
73
103
129
147
169
45
217
243
271
291 305
331
361
451

Supplementary Table S1. List of identified metabolites and the differences between control and parasitized leaves.

Retention time (min)	Compound name	Fragment ion (derivatized compound)[a]	p-value	Fold change	Identification NIST	STD
8.04	Phosphoric acid, 3TMS	298(100), 73(60), 313(18), 45(11)	0.13	0.73	○	
8.08	Glycerol, 3TMS	73(100), 147(78), 205(43), 117(32), 103(31)	0.18	1.47	○	○
8.73	Succinic acid, 2TMS	147(100), 73(52), 75(19), 148(16), 246(10)	3.0×10^{-4}	0.54	○	
9.25	Glyceric acid, 3TMS	73(100), 147(61), 189(31), 292(23), 102(17)	6.5×10^{-13}	0.53	○	
9.41	Fumaric acid, 2TMS	245(100), 73(57), 147(41), 143(23)	NM		○	
9.84	Serine, 3TMS	73(100), 204(95), 217(53), 100(27), 147(20)	NM		○	
10.43	Threonine, 3TMS	73(100), 117(48), 218(41), 57(22), 147(17)	NM		○	
12.16	Citramalic acid, 3TMS	73(100), 147(53), 247(41), 75(23), 115(15)	2.3×10^{-4}	0.33	○	
12.47	Malic acid, 2TMS	73(100), 147(53), 233(18)	0.05	0.87	○	
13.04	Erythritol, 4TMS	73(100), 147(57), 216(51), 103(34), 205(28)	1.3×10^{-13}	2.85	○	○
13.10	Aspartic acid, 3TMS	232(100), 73(98), 100(32), 147(22), 218(16)	NM		○	
13.73	Erythronic acid, 4TMS	73(100), 147(51), 292(24), 117(14), 103(11)	NM		○	
14.69	3-H-3-MP acid, 3TMS[b]	73(100), 147(47), 247(31), 115(18), 231(18)	NM		○	
15.27	Xylonic acid, 3TMS	73(100), 217(29), 147(27), 129(18), 102(11)	0.93	0.99	○	
16.00	Xylose, 4TMS, 1 MO	73(100), 103(77), 217(49), 307(32), 147(28)	0.018	1.50	○	
16.12	Arabinose, 4TMS	73(100), 103(88), 217(43), 307(29), 147(25)	3.0×10^{-8}	0.50	○	○
16.40	Ribose, 4TMS, 1 MO	73(100), 103(80), 217(44), 307(28), 147(24)	0.25	0.88	○	○
17.00	Xylitol, 5TMS	73(100), 217(64), 103(42), 147(40), 205(21)	0.015	1.49	○	○
17.21	Arabitol, 5TMS	73(100), 217(63), 103(44), 147(41), 205(22)	3.9×10^{-23}	11.39	○	○
17.29	Ribitol, 5TMS	73(100), 217(57), 147(43), 103(40), 205(27)	2.5×10^{-7}	2.43	○	○
17.92	Arabinofuranose, 4TMS	217(100), 73(92), 147(31), 232(10)	NM		○	○
18.01	Ribonic acid, 5TMS	73(100), 292(45), 147(36), 103(30), 217(29)	0.64	0.93	○	
18.23	Idonic acid, 4TMS	73(100), 147(36), 217(28), 103(24), 436(20)	8.2×10^{-4}	0.51	○	
18.82	Citric acid, 4TMS	73(100), 273(71), 147(59), 347(17), 375(14)	0.017	1.18	○	
19.91	Sorbose, 5TMS	73(100), 103(76), 217(52), 307(32), 147(24)	0.17	0.92	○	○
20.09	Mannose, 5TMS, 1 MO	73(100), 319(48), 205(41), 147(39), 160(26)	0.24	0.92	○	○
20.37	Fructose, 5TMS, 1 MO	73(100), 103(73), 217(53), 307(33), 147(24)	9.0×10^{-8}	0.46	○	○
20.65	Galactose, 5TMS, 1 MO	73(100), 319(49), 205(40), 147(39), 217(21)	1.0×10^{-10}	0.39	○	○
20.93	Mannitol, 6TMS	73(100), 319(56), 147(43), 205(39), 217(35)	1.7×10^{-17}	2.86	○	○
21.07	Sorbitol, 6TMS	73(100), 319(53), 147(42), 205(36), 103(29)	3.6×10^{-13}	0.35	○	○
22.10	Gluconic acid, 6TMS	73(100), 147(36), 333(28), 292(25), 103(15)	0.79	0.94	○	
23.39	myo-Inositol, 6TMS	73(100), 217(60), 305(58), 147(44), 318(31)	0.044	1.30	○	○
23.79	Glucose, 5TMS, 1 MO	73(100), 319(63), 205(38), 147(36), 103(21)	0.84	0.97	○	○
24.61	Linoleic acid, 1TMS	75(100), 73(97), 67(77), 81(70), 55(54)	NM		○	
24.70	Oleic acid, 1TMS	73(100), 117(94), 75(90), 129(64), 55(56)	1.54×10^{-6}	0.61	○	
25.06	Stearic acid, 1TMS	117(100), 73(75), 75(56), 341(55), 132(54)	0.061	0.81	○	
31.10	Sucrose, 8TMS	73(100), 361(88), 216(37), 147(25), 103(22)	0.32	0.82	○	○
32.84	Maltose, 8TMS	73(100), 361(86), 204(59), 147(34), 217(31)	0.007	1.70	○	○
33.97	Melibiose, 8TMS	204(100), 73(66), 217(29), 205(28), 361(27)	0.041	0.76	○	○

NIST: putatively identified using NIST database (match score over 80/100)
STD: identified using standard compound

Supplementary Table S2. Multivariate (VIP value > 1.00) and univariate (p-value < 0.05) statistical parameters of 26 marker metabolites with whole data (n=60) implying importance for discriminating control and parasitized groups.

Metabolites	VIP value	p-value
Erythritol	2.35	1.3×10^{-13}
Arabitol	2.34	3.9×10^{-23}
Galactose	1.89	1.0×10^{-10}
Citric acid	1.65	0.017
Maltose	1.50	0.007
myo-Inositol	1.47	0.044
Mannitol	1.45	1.7×10^{-17}
Arabinose	1.40	3.0×10^{-8}
Xylitol	1.40	0.015
Malic acid	1.39	0.05
Glucose	1.37	0.84
Ribitol	1.33	2.5×10^{-7}
Glycerol	1.28	0.18
Gluconic acid	1.26	0.79
Phosphoric acid	1.25	0.13
Fructose	1.22	9.0×10^{-8}
Mannose	1.08	0.24
Ribose	1.03	0.25
Sorbitol	1.01	3.6×10^{-13}
Sorbose	1.00	0.17
Succinic acid	<1.00	3.0×10^{-4}
Glyceric acid	<1.00	6.5×10^{-13}
Citramalic acid	<1.00	2.3×10^{-4}
Oleic acid	<1.00	1.54×10^{-6}
Melibiose	<1.00	0.041
Xylose	<1.00	0.018

Supplementary Table S3. Metabolic pathway enrichment results.

Metabolic pathway	Adjusted p-value	Total metabolites included	Matched metabolites	Matched/Total (%)
Pentose and glucuronate interconversions	2.08×10^{-04}	53	5	27.8
Galactose metabolism	9.06×10^{-04}	41	4	22.2
Fructose and mannose metabolism	0.015	48	3	16.7
Starch and sucrose metabolism	0.015	50	3	16.7
Glyoxylate and dicarboxylate metabolism	0.015	44	3	16.7
Biosynthesis of plant hormones	0.026	68	3	16.7
Citrate cycle (TCA cycle)	0.026	20	2	11.1
Biosynthesis of alkaloids derived from histidine and purine	0.056	35	2	11.1
Biosynthesis of phenylpropanoids	0.056	97	3	16.7
Biosynthesis of alkaloids derived from terpenoid and polyketide	0.084	48	2	11.1
Ascorbate and aldarate metabolism	0.084	47	2	11.1
Biosynthesis of alkaloids derived from shikimate pathway	0.102	138	3	16.7
Biosynthesis of alkaloids derived from ornithine, lysine and nicotinic acid	0.122	67	2	11.1
Amino sugar and nucleotide sugar metabolism	0.181	87	2	11.1
Biosynthesis of terpenoids and steroids	0.208	98	2	11.1

Supplementary Table S4. Enzymatic pathways about metabolic flows from AMN supplies (fructose, arabinose and galactose) to five sugar alcohols. Enzymes were designated as enzyme entry in KEGG pathway and modules. Enzymes and modules in PPP were colored as green.

Fructose	Ribitol	**Fructose** → (*EC:2.7.1.1*) Fructose-6P → (*M00007*) Ribose-5P → (*EC:2.7.1.15*) Ribose → (*EC:5.3.1.20*) Ribulose → (*EC:1.1.1.56*) Ribitol
	Arabitol	**Fructose** → (*EC:2.7.1.1*) Fructose-6P → (*M00007*) Ribose-5P → (*EC:2.7.1.15*) Ribose → (*EC:5.3.1.20*) Ribulose → (*EC:1.1.1.13*) Arabitol<br>Ribulose ↓ (*EC:5.3.1.4*) Arabinose → (*EC:1.1.1.21*) Arabitol<br>Fructose-6P ↓ (*EC:2.2.1.1*) Xylulose-5P → (*EC:2.7.1.17*) Xylulose → (*EC:1.1.1.287*, *EC:1.1.1.11*) Arabitol
	Erythritol	**Fructose** → (*EC:2.7.1.1*) Fructose-6P → (*EC:2.2.1.1*) Erythrose-4P → (*EC:2.7.1.27*) Erythritol
	Mannitol	**Fructose** → (*EC:2.7.1.1*) Fructose-6P → (*EC:1.1.1.17*) Mannitol-1P → (*EC:3.1.3.22*) Mannitol<br>**Fructose** → (*EC:1.1.1.67 or 1.1.1.138*) Mannitol
	Xylitol	**Fructose** → (*EC:2.7.1.1*) Fructose-6P → (*EC:2.2.1.1*) Xylulose-5P → (*EC:2.7.1.17*) Xylulose → (*EC:1.1.1.15*, *EC:1.1.1.10*, *EC:1.1.1.9*) Xylitol
Arabinose	Ribitol	**Arabinose** → (*EC:5.3.1.4*) Ribulose → (*EC:1.1.1.56*) Ribitol
	Arabitol	**Arabinose** → (*EC:1.1.1.21*) Arabitol
	Erythritol	**Arabinose** → (*EC:5.3.1.4*) Ribulose → (*EC:5.3.1.20*) Ribose → (*EC:2.7.1.15*) Ribose-5P → (*EC:2.2.1.2*, *EC:2.2.1.1*) Erythrose-4P → (*EC:2.7.1.27*) Erythritol
	Mannitol	**Arabinose** → (*EC:5.3.1.4*) Ribulose → (*EC:5.3.1.20*) Ribose → (*EC:2.7.1.15*) Ribose-5P → (*M00007*) Fructose-6P → (*EC:1.1.1.17*) Mannitol-1P → (*EC:3.1.3.22*) Mannitol
	Xylitol	**Arabinose** → (*EC:5.3.1.4*) Ribulose → (*EC:5.3.1.20*) Ribose → (*EC:2.7.1.15*) Ribose-5P → (*EC:2.2.1.2*, *EC:2.2.1.1*) Xylulose-5P → (*EC:2.7.1.17*) Xylulose → (*EC:1.1.1.15*, *EC:1.1.1.10*, *EC:1.1.1.9*) Xylitol
Galactose	*Leloir pathway*	**Galactose** → (*EC:2.7.1.6*) Galactose-1P → (*EC:2.7.7.12*) Glucose-1P → (*EC:5.4.2.5*, *EC:5.4.2.2*) Glucose-6P
	Ribitol	**Galactose** → (*Leloir pathway*) Glucose-6P → (*M00006*) Ribulose-5P → (*EC:2.7.1.16*) Ribulose → (*EC:1.1.1.56*) Ribitol
	Arabitol	**Galactose** → (*Leloir pathway*) Glucose-6P → (*M00006*) Ribulose-5P → (*EC:2.7.1.16*) Ribulose → (*EC:1.1.1.13*) Arabitol
	Erythritol	**Galactose** → (*Leloir pathway*) Glucose-6P → (*EC:5.3.1.9*) Fructose-6P → (*EC:2.2.1.1*) Erythrose-4P → (*EC:2.7.1.27*) Erythritol
	Mannitol	**Galactose** → (*Leloir pathway*) Glucose-6P → (*EC:5.3.1.9*) Fructose-6P → (*EC:1.1.1.17*) Mannitol-1P → (*EC:3.1.3.22*) Mannitol
	Xylitol	**Galactose** → (*Leloir pathway*) Glucose-6P → (*M00006*) Ribulose-5P → (***EC:5.1.3.1***) Xylulose-5P → (*EC:2.7.1.17*) Xylulose → (*EC:1.1.1.15*, *EC:1.1.1.10*, *EC:1.1.1.9*) Xylitol
